# Cellular and Gene Expression Response to the Combination of Genistein and Kaempferol in the Treatment of Mucopolysaccharidosis Type I

**DOI:** 10.3390/ijms23031058

**Published:** 2022-01-19

**Authors:** Magdalena Węsierska, Anna Kloska, Diego L. Medina, Joanna Jakóbkiewicz-Banecka, Magdalena Gabig-Cimińska, Marta Radzińska, Marta Moskot, Marcelina Malinowska

**Affiliations:** 1 Department of Medical Biology and Genetics, Faculty of Biology University of Gdańsk, Wita Stwosza 59, 80-308 Gdańsk, Poland; magdalena.wesierska@phdstud.ug.edu.pl (M.W.); anna.kloska@ug.edu.pl (A.K.); joanna.jakobkiewicz-banecka@ug.edu.pl (J.J.-B.); magdalena.gabig-ciminska@ug.edu.pl (M.G.-C.); marta.radzinska@purebiologics.com (M.R.); 2 Telethon Institute of Genetics and Medicine (TIGEM), Via Campi Flegrei 34, 80078 Naples, Italy; medina@tigem.it; 3 Medical Genetics Unit, Department of Medical and Translational Science, Federico II University, Via Pansini 5, 80131 Naples, Italy; 4 Laboratory of Molecular Biology of Human Skin Diseases, Institute of Biochemistry and Biophysics, Polish Academy of Sciences, Kładki 24, 80-822 Gdańsk, Poland

**Keywords:** flavonoids, GAG metabolism, gene expression, mucopolysaccharidosis type I

## Abstract

Flavonoids are investigated as therapeutics for mucopolysaccharidosis, a metabolic disorder with impaired glycosaminoglycan degradation. Here we determined the effects of genistein and kaempferol, used alone or in combination, on cellular response and gene expression in a mucopolysaccharidosis type I model. We assessed the cell cycle, viability, proliferation, subcellular localization of the translocation factor EB (TFEB), number and distribution of lysosomes, and glycosaminoglycan synthesis after exposure to flavonoids. Global gene expression was analysed using DNA microarray and quantitative PCR. The type and degree of flavonoid interaction were determined based on the combination and dose reduction indexes. The combination of both flavonoids synergistically inhibits glycosaminoglycan synthesis, modulates TFEB localization, lysosomal number, and distribution. Genistein and kaempferol in a 1:1 ratio regulate the expression of 52% of glycosaminoglycan metabolism genes. Flavonoids show synergy, additivity, or slight antagonism in all analysed parameters, and the type of interaction depends on the concentration and component ratios. With the simultaneous use of genistein and kaempferol in a ratio of 4:1, even a 10-fold reduction in the concentration of kaempferol is possible. Flavonoid mixtures, used as the treatment of mucopolysaccharidosis, are effective in reducing glycosaminoglycan production and storage and show a slight cytotoxic effect compared to single-flavonoid usage.

## 1. Introduction

Flavonoids are polyphenolic compounds synthesised in plants as secondary metabolites and are qualitatively and quantitatively one of the largest known groups of natural products. In addition, they are the most vital phytochemicals in the human diet and are of great general interest due to their diverse bioactivity and considerable biological and health benefits [1,2]. Epidemiological and nutritional data have demonstrated that natural flavonoids play an important role in the prevention and management of many diseases, such as bacterial and viral infections, cancer, cardiovascular diseases, inflammation, and even some genetic diseases [3,4,5,6]. 

These compounds have also been extensively investigated as potential therapeutic agents for mucopolysaccharidosis (MPS), which is a genetic metabolic disorder resulting from impaired degradation of glycosaminoglycans (GAGs) due to deficiencies in lysosomal enzymes involved in GAG catabolism. Progressive intra- and extracellular accumulation of GAGs leads to widespread tissue and organ dysfunction (including heart, respiratory system, bones, joints, and, in some MPS types, central nervous system) and reduced life expectancy [7].

One of the therapeutic strategies for MPS, which is called substrate reduction therapy (SRT), is based on small molecular inhibitors that can impair the intracellular synthesis of compounds that cannot be degraded in lysosomes to prevent or slow down lysosomal storage. Most studies on the biological effects of flavonoids in SRT focus on genistein (G), which is an isoflavone naturally occurring in soybeans, that efficiently inhibits GAG synthesis in both MPS and normal human fibroblast cultures [8]. In addition, it reduces total GAG content and lysosomal compartment size in visceral organs of MPS II [9] and MPS IIIB mice [10], as well as corrects the neuropathology and behaviour of MPS IIIB mice following long-term treatment [11]. Kaempferol (K), the other compound that belongs to flavonoids, also efficiently inhibits GAG synthesis or reduces GAG storage in fibroblast culture models of different MPS types [12,13]. Moreover, combinations of two or more flavonoids decrease GAG synthesis [12] and GAG content [13] more efficiently than the equivalent amounts of these compounds alone. These studies strongly suggest that the interactions of flavonoids applied in a mixture should be taken into consideration while developing a therapeutic strategy for MPS that is based on natural compounds, such as flavonoids.

On the market, flavonoids are most widely available as extracts or mixtures of different compositions; thus, interactions between these components should be taken into consideration while assessing the benefit of such products. Isobolographic analysis is a method for rigorous evaluation of the type and degree of interactions between two active compounds applied in a mixture. It provides a basis for evaluating whether a biological response induced by mixtures of chemicals is additive, synergistic, or antagonistic. In general, if the response to two compounds in a mixture improves the response compared to that obtained for each of the compounds acting alone, the effect is synergistic; if the response remains unchanged, the effect is additive; and if the response is reduced, the effect is antagonistic [14]. The main reason for using two or more compounds in combination is to increase the efficacy of the treatment while decreasing its toxicity. The combination of flavonoids used for the treatment of mucopolysaccharide disorders should only be considered if it can effectively reduce GAG production and storage, as well as exhibit no or minor cytotoxic effects. 

In our present study, we characterised the type and degree of interactions among genistein and kaempferol using isobolographic analysis. We investigated the effects of these flavonoids, exerted in two-component mixtures, regarding the cytotoxicity, cell cycle and viability, proliferation, and GAG synthesis in a cell culture model to confirm possible advantages of using flavonoid combinations over a single flavonoid for the treatment of MPS. Further intrinsic analyses employing global gene expression and ontology enrichment study and estimation of lysosomal biogenesis transcription factor nuclear translocation (TFEB) and lysosomal number enabled estimation of the potential in vitro impacts of flavonoid mixtures.

## 2. Results

### 2.1. Single Flavonoid Treatment Affects Cell Proliferation and Inhibits GAG Synthesis in Murine Fibroblasts

Depending on the different solubilities of the flavonoids used in our study, the range of concentrations tested in cytotoxicity, cell viability, and proliferation assays was 1–300 µM for G and 1–150 µM for K. The kinetics of GAG synthesis were estimated for all flavonoids at concentrations of 30, 60, and 120 µM. The effect of flavonoids in all assays was determined from dose–response curves and was expressed as half-maximal effective concentration (EC50) values. Generally, G and K were non-cytotoxic in the tested range of concentrations, but both affected cell proliferation with similar potencies (EC50 values 47 ± 19.3 for G and 55 ± 23.00 for K). All tested flavonoids significantly inhibited GAG synthesis in a dose-dependent manner (data not shown) and with potencies similar to each other. Interestingly, the effective reduction of GAG synthesis by half was achieved at flavonoid concentrations about 2- or 3-fold lower than those that affected cell proliferation (EC50 values 25 ± 5.2 for G and 29 ± 2.0 for K).

### 2.2. Different Impact of Final Concentration and Composition of Flavonoids in the Mixture on Cell Viability, Proliferation, and GAG Synthesis of Murine Fibroblasts 

**Figure 1:**
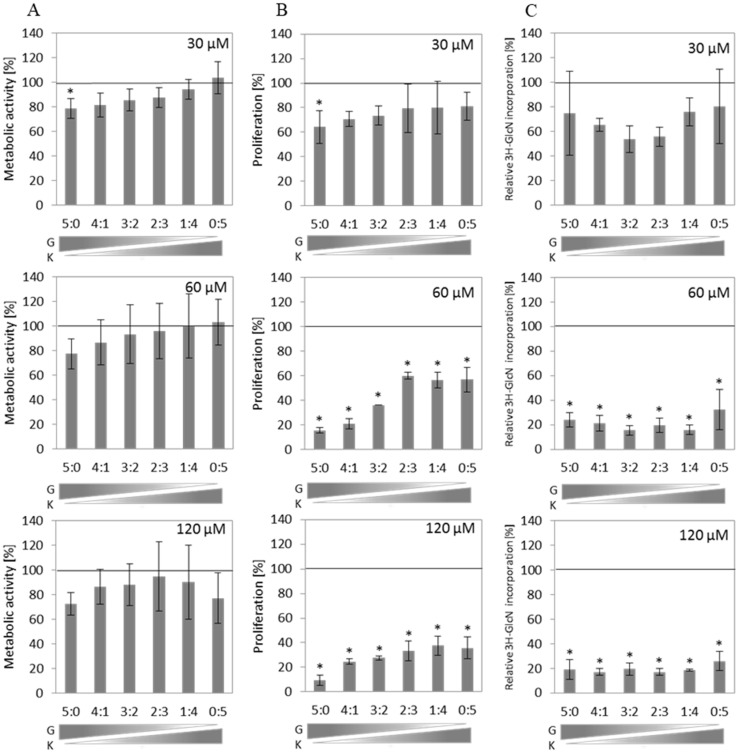
Effects of genistein and kaempferol in dose ratios of 5:0; 4:1; 3:2; 2:3; 1:4; and 0:5 at final concentrations of 30 µM, 60 µM, and 120 µM on fibroblast metabolic activity (**A**) measured by MTT assay after 24 h exposition, proliferation (**B**) measured by BrdU incorporation assay, and GAG synthesis (**C**) measured with a D-[1-3H]-glucosamine incorporation assay after 48 h exposition. The black line (100%) corresponds to metabolic activity in control cells (cell culture treated with 0.05% DMSO). Bars represent the average ± SD obtained for two different cell cultures from two different experiments in each case. An asterisk (*) indicates statistically significant differences when compared to control cells (ANOVA with Tukey’s post hoc, *p* < 0.05).

Cell viability of murine fibroblasts, measured as metabolic activity via MTT assay, was slightly but not statistically significantly decreased by different flavonoid mixtures in all tested conditions (Figure 1A). However, cell proliferation assessed by BrdU incorporation was slightly altered at a total concentration of 30 µM and significantly reduced by flavonoid mixtures at 60 µM and 120 µM compared to the untreated control (Figure 1B). The inhibition level of this process also depends on the G content in the mixture. Replacement of G with increasing amounts of K resulted in the reduction of the anti-proliferative activity of the flavonoid mixtures (Figure 1B). Kinetics of GAG synthesis was estimated by measurement of incorporation of ^3^H-GlcN after 48 h incubation of murine primary fibroblasts with tested compounds. The level of GAG synthesis was significantly reduced in all mixtures and ratios used in a total concentration of at least 60 µM (Figure 1C). 

### 2.3. Flavonoids in the Mixtures Show all Types of Interactions for the Kinetics of GAG Synthesis in Murine Primary Fibroblasts

**Figure 2:**
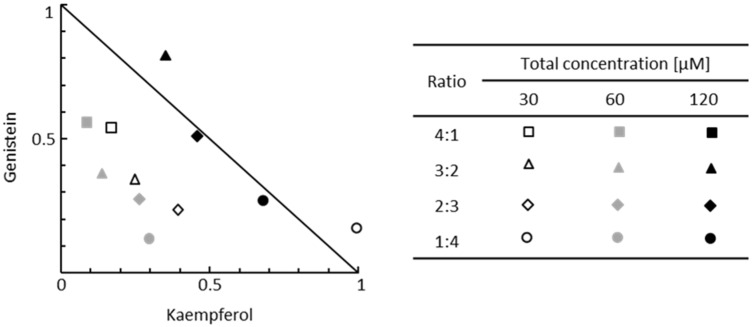
Normalised isobologram for two-component mixtures of genistein and kaempferol at specified ratios. GAG synthesis was estimated after 48 h incubation of fibroblasts exposed to mixtures of flavonoids by measurement of incorporation of D-[1-3H]-glucosamine. The solid line denotes the theoretical additivity in the reduction of GAG synthesis by given flavonoids.

Interactions between flavonoids used in a mixture regarding the process of GAG synthesis have not been investigated to date; thus, we aimed to assess the type of interactions between G and K using the isobolographic analysis of two-component flavonoid mixtures. The kinetics of GAG synthesis was estimated by measuring the incorporation of 3H-GlcN after 48 h incubation of murine primary fibroblasts with fixed-ratio mixtures of flavonoids (Figure 1C). Experimental values of GAG synthesis levels obtained for four fixed-ratio flavonoid combinations were plotted against the theoretical additivity line marked for values of the GAG synthesis levels obtained for single flavonoid treatments at specified final concentrations (Figure 2). When the plotted point falls below the additivity line, the components in a mixture act synergistically, and when the plotted point is above the additivity line, the components of the tested mixture act antagonistically. In our experimental setup, GAG synthesis was significantly reduced in all mixtures and ratios used with a total concentration of at least 60 µM (Figure 1C). The type of interactions between flavonoids was quantified according to calculated combination index (CI) values (Table 1). We observed the whole spectrum of interactions between G and K in two-component mixtures (Figure 1C and Table 1). At the final concentration of 60 µM, the activity of G was strongly enhanced when combined with K (synergism). Furthermore, we calculated the dose reduction index (DRI) to evaluate the fold of a dose reduction for G and K when used in combinations to achieve inhibition of GAG synthesis comparable to those obtained with a single flavonoid. Substantial dose reduction can be achieved when flavonoids are used in two-component mixtures (Table 1). In mixtures with the final concentration of 30 μM or 60 μM flavonoids, the dose of G in the G–K combination required to achieve the same level of GAG synthesis inhibition as for G alone was reduced by up to 8-fold. For the same combination, the dose of K was also decreased by up to 10-fold to achieve the same effect as K alone. The fold of dose reduction varied depending on the ratio of components used in a mixture. In the case of antagonistic or additive interactions between G and K, the dose reduction of the components in a mixture was rather unfavourable or not prominent. 

### 2.4. Different Impact of Final Concentration and Composition of Genistein and Kaempferol in Two-Component Mixtures on the Cell Cycle

Murine fibroblast cultures were derived from tail skin explants. Therefore, to assess the effect of the tested compounds on the cell cycle, their full physiological characterization was necessary. Cells seeded with an initial number of 5 × 10^4^ per well of a standard 6-well plate exhibited the log (exponential) phase of growth between days 1 and 5 after subculture (Figure S1), with the doubling time estimated at 18.11 h. Thus, all experiments assessing the cell cycle profile were performed between days 2 and 5 after subculture. 

To determine the full characteristics of the cell cycle phases, we measured the percentage of cells in the G0/G1, S, and G2/M phases using an assay based on the intercalation of propidium iodide (PI) into DNA. The measurement of the PI-based staining of DNA content using the Muse^®^ Cell cycle assay allows one to discriminate and indicate the percentage of cells in each cell cycle phase. During the log phase of growth, the population of control cells (not exposed to flavonoids) consisted of over 50% of cells in the G0/G1 phase; this number significantly increased with time up to 67% after 3 days of culture, while the number of cells in the S phase significantly decreased over the same time range. Furthermore, the number of cells in G2/M did not change in a statistically significant manner in the studied time (Figure S1). 

Exposure to G or K resulted in significant cell cycle arrest in the G2/M phase; the effect was visible after 24 h and lasted until 48 h after treatment (Figure S2). Cell cycle inhibition depends on the flavonoid concentrations used; 30 µM for G and 60 µM for K were sufficient to disturb the cell cycle, as the number of cells in the G2/M phase increased as early as 24 h after treatment. 

**Figure 3:**
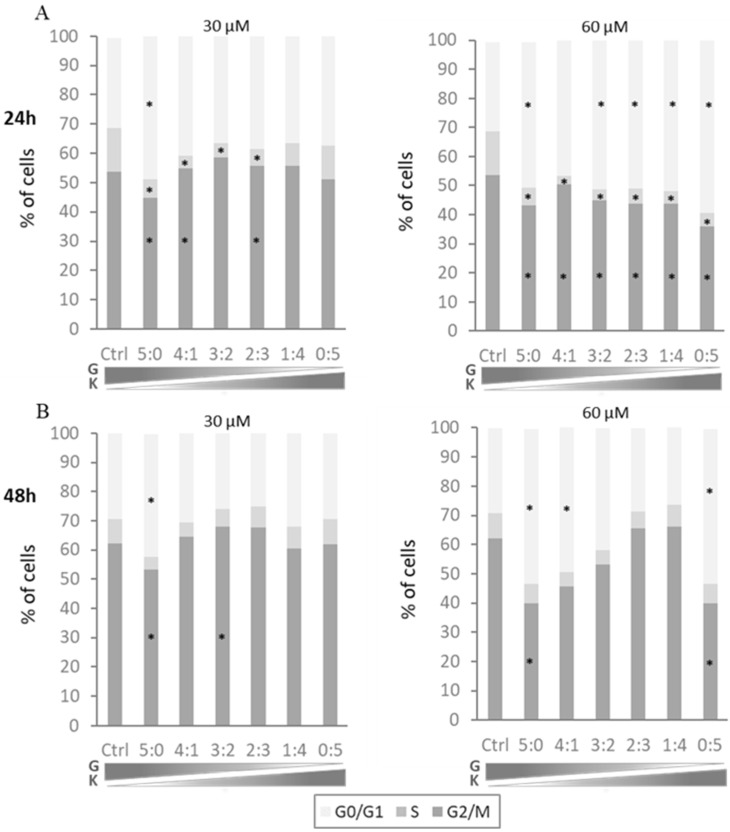
The cell cycle of mouse fibroblasts after 24 h (**A**) or 48 h (**B**) exposure to mixtures of genistein and kaempferol at 30 µM and 60 µM final concentrations. Each two-component mixture was tested at ratios of 5:0, 4:1, 3:2, 2:3, 1:4, and 0:5. Ctrl—control cells treated with 0.1% DMSO; K—kaempferol; G—genistein. Data are presented as means of at least two independent biological repetitions. Most of the SD values are in the range of 1% to 3%; therefore, they are not shown in the charts. An asterisk (*) indicates statistically significant differences when compared to control cells (ANOVA with Tukey–Kramer HSD post hoc test, *p* < 0.05 or less).

The effect of two-component mixtures of flavonoids on the cell cycle profile of murine fibroblasts was assessed after 24 and 48 h exposure to genistein with kaempferol at final concentrations of 30 µM and 60 µM and proportions of components at 4:1, 3:2, 2:3, and 1:4. Mixtures of genistein with kaempferol at a total concentration of 30 μM significantly affected the number of cells in the S phase when compared to untreated controls. The population of DNA synthesizing cells was smaller following 24 h exposure to the tested flavonoids at almost all tested ratios, while the cells exposed to the tested compounds for 24 h at a total concentration of 60 μM showed significant disturbances of the cell cycle in all phases (Figure 3A). Cells exposed to the tested flavonoid mixtures for 48 h for both total concentrations presented with less pronounced disturbances. Furthermore, any significant reduction of the cell numbers in the G2/M phase or increase in the G0/G1 phase occurred only for single flavonoids or mixtures with a predominance of genistein (Figure 3B). 

### 2.5. TFEB Subcellular Localisation, and Lysosomal Number and Distribution Are Modulated by Flavonoids

**Figure 4:**
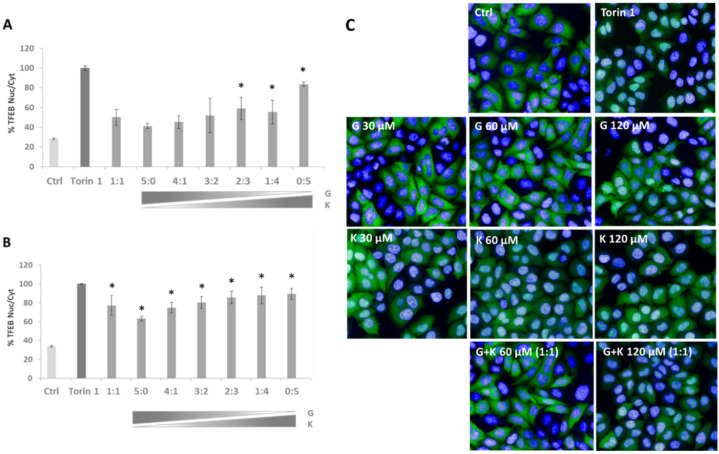
TFEB nuclear translocation in TFEB-FLAG HeLa cells after 24 h exposure to genistein (G) and kaempferol (K) mixtures at final concentrations of 60 μM (**A**) and 120 μM (**B**). Each two-component mixture was tested at ratios of 5:0, 4:1, 3:2, 2:3, 1:4, and 0:5. Ctrl—control cells treated with 0.1% DMSO; G—genistein; K—kaempferol. Visualisation of TFEB nuclear translocation was performed for single tested flavonoids at final concentrations of 30 μM, 60 μM, and 120 μM, and 1:1 mixtures at final concentrations of 60 μM or 120 μM (**C**). Torin 1 was employed as the positive control. Representative images were taken from fields containing 50–100 cells, each from six independent experiments. Nucleus stained with DAPI (blue); TFEB immunostained with FLAG (green). All values are means ± S.D. (Student’s *t*-test (unpaired); * *p* < 0.005)). An asterisk (*) indicates statistically significant differences when compared to control cells.

Based on our previous investigations with flavonoids, we determined if there were variations in the modulation of transcription factor EB (TFEB) nuclear translocation upon the applications of selected flavonoid mixtures. Upregulation of TFEB translocation in TFEB-FLAG HeLa cells was observed in all treatment conditions in reference to DMSO-treated controls, and the effect was superior in mixtures of final flavonoid concentrations of 60–120 µM (Figure 4B). The highest TFEB nuclear translocation was denoted for 60 µM K, 120 µM K (represented as the ratio of 0:5 in Figure 4A,B, respectively), and 120 µM final concentration mixture of G and K in the ratio 1:4 (Figure 4B), i.e., with 84%, 89%, and 87% of translocation level upon exposure to Torin 1 considered as the positive control. Significant differences were observed between individual flavonoid treatments. In the case of 60 µM single flavonoid final concentration, TFEB nuclear translocation was 42% more effective in K (Figure 4A, represented as the ratio 0:5) than in samples treated with G (Figure 4, panel A, represented as the ratio 5:0), while at the 120 µM single flavonoid final concentration, translocation was 26% higher in K (Figure 4B, represented as the ratio 0:5) than in samples treated with G (Figure 4B, represented as the ratio 5:0), respectively. For particular mixture composition ratios, alterations in TFEB translocation were rather minor (~10%).

**Figure 5:**
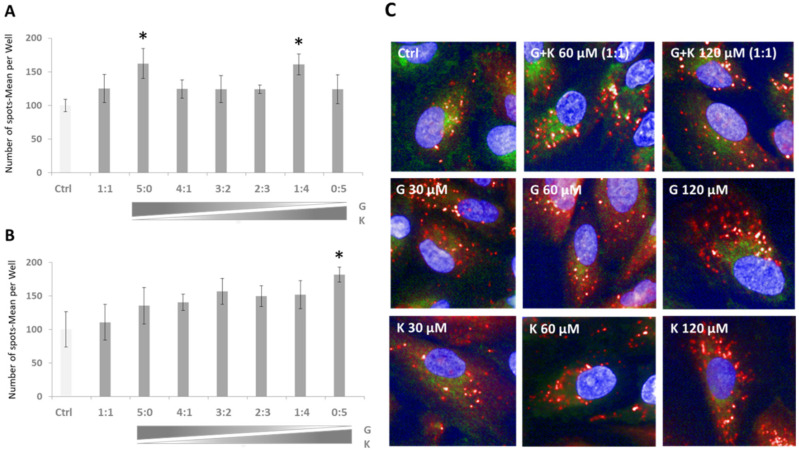
Lysosome number estimation with LysoTracker™ Red DND-99 upon 24 h exposure to G and K mixtures at final concentrations of 60 μM (**A**) and 120 μM (**B**). Each two-component mixture was tested at ratios of 5:0, 4:1, 3:2, 2:3, 1:4, and 0:5. Ctrl—control cells treated with 0.1% DMSO; G—genistein; K—kaempferol. Exemplary pictures are presented for single tested flavonoids at final concentrations of 30 μM, 60 μM, and 120 μM and 1:1 G and K mixtures at final concentrations of 60 μM and 120 μM (**C**). An asterisk (*) indicates statistically significant differences when compared to control cells.

As TFEB is a master regulator of lysosomal biogenesis, the next step in the assessment of the impact of flavonoid mixtures was to estimate whether there is a modulation of the number of lysosomes upon flavonoid action. For this reason, the LysoTracker™ Red DND-99 assay was implemented in ARPE-19 cells. An increase in the number of lysosomes by 72% was found in cells treated with 120 µM K (Figure 5B, designated as the ratio 0:5), 59% in 60 µM (Figure 5A, represented as the ratio 5:0), and of 57% in the 60 µM final concentration mixture of G and K at the ratio of 1:4 (Figure 5A) when compared to DMSO-treated control cells, respectively. Furthermore, the number of lysosomes was increased in all tested conditions. 

One of the cell metabolic state parameters is lysosomal distribution. Lysosomal positioning performed by immunofluorescence analysis displayed peripheral localisation of lysosomes upon treatment with flavonoids when compared to untreated controls (Figure 5C). This situation is rather typical for high nutrient, low autophagosome synthesis and low autophagosome-lysosome fusion states.

### 2.6. Kaempferol Has a Prevalent Impact on Murine Primary Fibroblasts Global Gene Expression in Flavonoid Mixtures

To determine the impact of the tested flavonoids on global gene expression analysis with the Clariom S Affymetrix (Thermo Fisher Scientific, Waltham, MA, USA), mouse assays were performed for MPS I murine fibroblasts upon treatment with 60 and 120 μM G, 60 and 120 μM K, and 60 and 120 μM final concentration mixtures of both flavonoids. To correlate the impact of genistein and kaempferol mixtures with single flavonoid treatments, a 1:1 ratio of both flavonoids was employed. The composition of equal compounds’ concentrations enables extensive analysis of the intermediate influence of such a combination. Based on this knowledge, further intrinsic analysis applying versatile flavonoids ratios could be conducted. 

**Figure 6:**
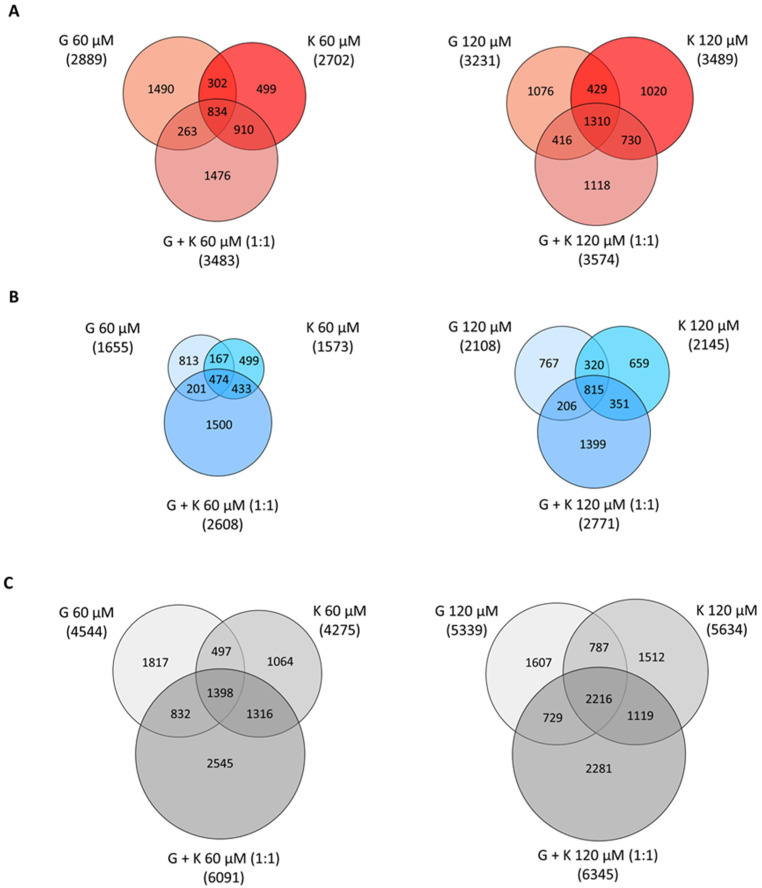
Venn diagrams illustrating the number of genes upon microarray analysis with expression modulated in response to genistein and kaempferol at 30 µM, 60 µM, and 120 µM and to flavonoid mixtures at final concentrations of 60 µM and 120 µM. Data presented on the red graphs (panel (**A**)) demonstrate the number of transcripts with upregulation, on the blue graphs (panel (**B**)) demonstrate the number of transcripts with downregulation, and on the grey graphs (panel (**C**)) demonstrate the transcripts with expression modulated upon flavonoid treatment, respectively. G—genistein, K—kaempferol; G + K—1:1 ratio of genistein and kaempferol mixture.

Fold changes less than 0.7 and more than 1.3 in reference to control experimental conditions (0.05 and 0.1% DMSO treatment for single flavonoids and mixtures, respectively) was assumed as positively and negatively regulated, respectively. One of the results of the performed analysis was the observation that flavonoids induce dose- and time-dependent alterations in transcript profiles in vitro (Figure 6). The highest number of genes with modulated expression, 6345 transcripts (out of >22,100 genes, >150,300 transcripts designated by >221,900 microarray probes), was denoted for the 1:1 G and K mixture at a final concentration of 120 µM. This stands for almost 30% of the analysed genes and sequences of the murine genome. In addition, the highest numbers of upregulated and downregulated genes were recorded in this condition, namely 3574 and 2771, respectively. 

It has to be mentioned that the higher concentration of flavonoids in a 1:1 mixture had a minor effect on the number of genes with modulated expression—only 2.5%, 5.9%, and 4.0% more genes were upregulated, downregulated, and regulated in total, respectively, for the 120 µM final concentration compared to the G and K 1:1 60 µM mixture. For treatment with single flavonoids, an increase in the number of genes with upregulated, downregulated, and in total regulated expression was 10.6%, 21.5%, and 14.5%, respectively, for 120 µM G compared to 60 µM G and 22.6%, 26.7%, and 24%, respectively, for 120 µM K treatment compared to 60 µM K. 

Microarray analysis revealed that the number of genes with modulated expression was comparable for corresponding concentrations of G and K applied separately. However, it varied significantly between the G and K 1:1 mixture applied in the final 60-µM concentration and both flavonoids tested separately. Murine fibroblast cultivation with the addition of the 60-µM mixture of G and K in a ratio of 1:1 resulted in an increase by 18%, 37%, and 25% in the number of upregulated, downregulated, and regulated in total genes, respectively, compared to G used alone in a final 60-µM concentration. When compared to 60-µM K used alone, exposure to the 60-µM mixture of G and K in a 1:1 ratio increased by 23%, 40%, and 30% the number of genes with upregulated, downregulated, and regulated in total expression, respectively. In the case of the final 120-µM concentration of flavonoids, for a 1:1 mixture of G and K, the number of genes was by increased by 10% for upregulated, 24% for downregulated, and 16% for genes regulated in total, compared to treatment with G alone, while the number of genes with upregulated, downregulated, and regulated in total expression increased only by 3%, 23%, and 11%, respectively, after the mixture compared to the effect of 120-µM K used alone.

For all tested conditions, the upregulated genes presented 60% among all modulated transcripts, and the approximate 40% left constituted the downregulated genes. The number of modulated genes was comparable for that using treatment with G and K alone between both concentrations tested. The number of modulated transcripts common for treatment with K alone and the 1:1 ratio of the G and K mixture was greater in comparison to the respective gene group for treatment with G alone, as well as the 1:1 ratio of G and K mixture, for each analysis performed (Figure 6). 

### 2.7. High Impact of Genistein and Kaempferol Mixture on Modulation of Expression of Genes Involved in Glycosaminoglycan Metabolism Pathway 

**Figure 7:**
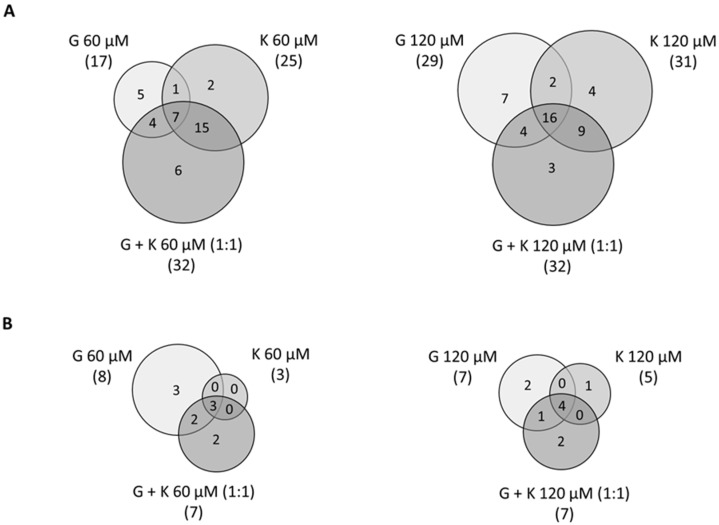
Venn diagrams illustrating the number of modulated genes upon microarray analysis coding for GAG synthesis (**A**) and degradation (**B**) proteins in response to genistein, kaempferol, and genistein and kaempferol mixture at final concentrations of 60 µM and 120 µM. G—genistein; K—kaempferol; G + K—1:1 ratio of genistein and kaempferol mixture.

Among the 75 genes related to GAG metabolism (57 genes of synthesis and 18 of degradation), around 40% were found to be significantly differentially expressed (FC less than 0.7 and more than 1.3 in reference to control experimental conditions, 0.1% DMSO treatment) in murine MPS I fibroblasts in all tested conditions, as revealed by the microarray analysis (Figure 7). Interestingly, for both GAG synthesis and degradation, as well as in total for GAG metabolism, conditions with the highest number of modulated genes were 60 µM and 120 µM of a 1:1 mixture of G and K, with 39 modulated genes of GAG metabolism (out of 75 genes, 52%), 32 of GAG synthesis (out of 57 genes, 56%), and 7 of GAG degradation (out of 18 genes, 39%). The conditions with the least numerous modulated transcripts were 60 µM G with 25 modulated genes of GAG metabolism (33%), 120 µM K with 16 modulated genes of GAG synthesis (28%), and 60 µM K with 3 modulated genes of GAG degradation (17%). Microarray expression analysis of transcripts coding for proteins involved in GAG metabolism revealed consistent profiles, and many regulated genes were common for all tested conditions (i.e., *B4galt4, Chst11, Chst12, Csgalnact1, Hs3st3a1, Hs6st2,* and *Hs6st3* for GAG synthesis and *Hgsnat, Hyal1,* and *Naglu* for GAG degradation (Table S1).

Real-time qRT-PCR analysis of selected genes coding for proteins involved in GAG metabolism pathways, in samples treated with 120 µM G, 120 µM K, and 120 µM 1:1 mixture of both flavonoids, confirmed the results of the microarray examination and revealed a consistent profile of gene expression modulation between the tested flavonoids and their 1:1 mixture (Table 2). Real-time qRT-PCR expression was performed in reference to the housekeeping control genes *B2m, Gapdh*, and *Hmbs* for all tested conditions.

For upregulated genes coding for GAG synthesis proteins, the fold change varied significantly between conditions with particular flavonoids or 1:1 mixtures (Table 2). Positively modulated transcripts were represented by *Csgalnact1* (coding for GAG chain initiation protein), *B4galt4, Chsy1, Extl3* (coding for chain elongation proteins), and *Chst2* (coding for chain modification proteins). The downregulated transcripts were *B3gnt2*, *B4galt1*, and *Ext1* (coding for proteins involved in GAG chain elongation), as well as *Dse* and *Ndst2* (coding for GAG chain modification proteins) (Table 2). Analysis performed for the GAG degradation pathway revealed upregulation of expression of genes coding for proteins involved in the degradation of heparan sulphate (HS) (*Hgsnat, Naglu,* and *Sgsh*), keratan sulphate (KS) (*Hexb*), chondroitin sulphate (CS), and hyaluronan (*Hyal1*) (Table 2). Abnormalities connected with the expression of GAG degradation enzymes occur in MPS IIIC, MPS IIIB, and MPS IIIA (HS degradation); Sandhoff disease and gangliosidosis (KS degradation); and MPS IX (CS degradation), respectively. 

According to microarray analysis of the transcripts related to GAG metabolism, most upregulated gene expression values were denoted for *Agrn, Gpc2*, and *Sulf2*. Most downregulated transcripts were estimated for *Angpt1, Sdc3*, and *Sulf1* (Table S1).

### 2.8. Genistein at High Concentration Has a Significant Impact on the Expression of Genes Coding for Cell Cycle Factors in Murine Primary Fibroblasts 

**Figure 8:**
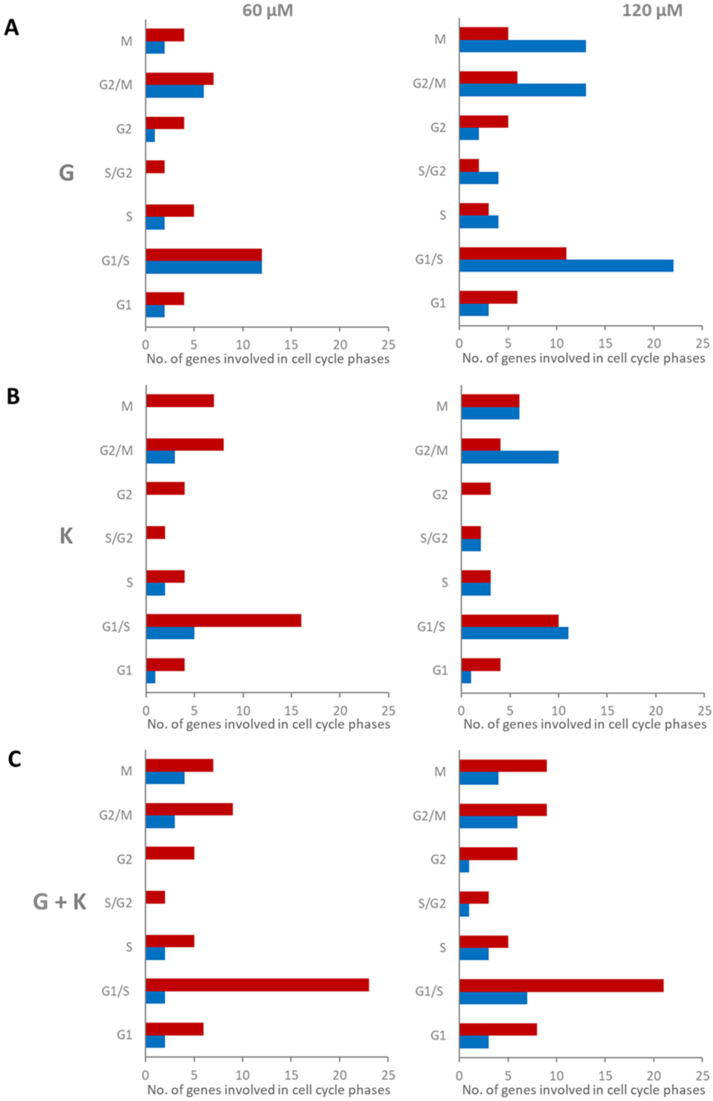
Gene Ontology (GO) analysis of murine MPS I fibroblast genes coding for factors involved in particular cell cycle phases, modulated upon 24 h treatment with genistein (**A**), kaempferol (**B**), and genistein and kaempferol 1:1 mixture (**C**) at final concentrations of 60 μM and 120 μM. Upregulated genes are presented as red columns, while downregulated genes are presented as blue columns. G—genistein, K—kaempferol, G + K—genistein + kaempferol 1:1 mixture.

To assess the precise mode of flavonoids cell cycle phase regulation, microarray analysis was performed for a list of 96 genes coding for proteins developed based on the cell cycle pathway in Cell Cycle SuperPath (based on KEGG and BioSystems) (Table S2). Fold changes less than 0.7 and more than 1.3 in reference to control experimental conditions (0.1% DMSO treatment) were assumed as positively and negatively regulated, respectively. Most genes with modulated expression, coding for cell cycle pathway factors, were assigned for 120 μM G treatment, 58 genes (60.4% of all analysed for cell cycle, 18 upregulated and 40 downregulated), and for the 120 μM and 60 μM 1:1 mixture of flavonoids, 49 (51%, 33 upregulated and 16 downregulated) and 44 (45.8%, 34 upregulated and 10 downregulated) modulated genes, respectively (Figure 8). When the analysis was more specifically focused on the type of gene expression regulation, it was noted that the 60 and 120 μM 1:1 flavonoid mixtures resulted in the most numerous genes with upregulated expression for all cell cycle phases: 6 and 8 out of 14 for G1, 23 and 21 out of 51 for G1/S, 5 out of 13 for S, two and three out of seven for S/G2, five and six out of seven for G2, 9 out of 29 for G2/M, and 7 and 9 out of 29 for M, respectively. On the contrary, most genes with downregulated expression for all cell cycle phases were observed for treatment with 120 μM G: three for G1, 22 for G1/S, four for S, four for S/G2, two for G2, 13 for G2/M, and 13 for M. G1/S, G2/M and M were the cell cycle phases with the most numerous modulated genes. Furthermore, the most significant differences in the number of modulated genes between experimental conditions were also observed for these phases, especially between 60 and 120 μM G.

### 2.9. Flavonoids Have a Positive Impact on the Vacuolar Organisation and Transport According to Gene Ontology (GO) Analysis

**Figure 9:**
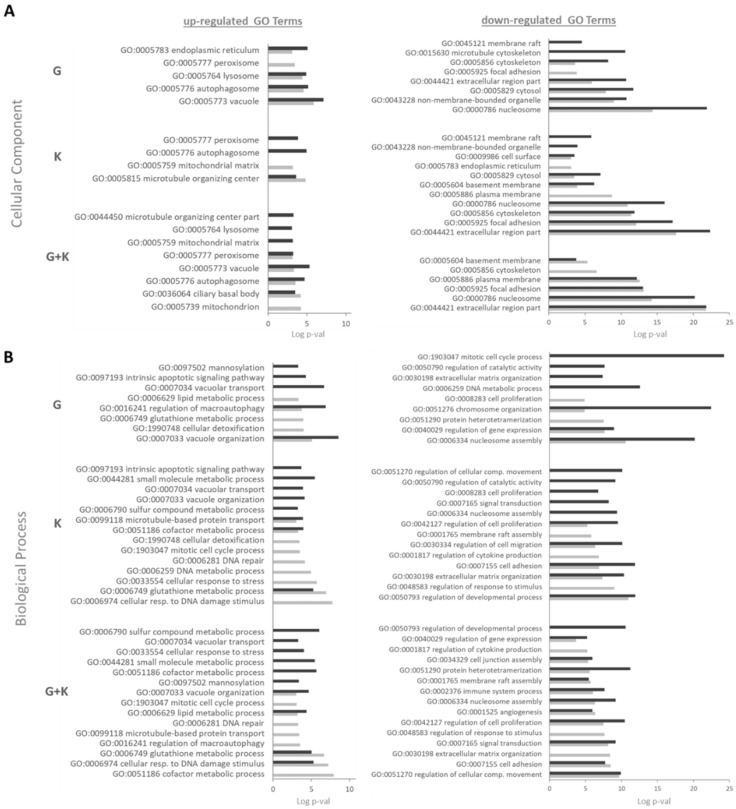
Gene Ontology (GO) analysis of the biological process (BP) (**A**) and cellular compartment (CC) (**B**). Columns on the graph present the log *p*-value, factor discriminative for GO terms describing biological processes and cellular components most enriched upon 60 μM of a single flavonoid or 1:1 mixture (pale shaded) and 120 μM of a single flavonoid or 1:1 mixture (dark shaded). G—genistein, K—kaempferol, G + K—genistein + kaempferol 1:1 mixture.

Detailed Gene Ontology (GO) analysis of transcripts modulated upon 60 and 120 μM G, 60 and 120 μM K, and 60 and 120 μM G and K 1:1 mixtures depicted the autophagosome (GO:0005776) and peroxisome (GO:0005777) as being mainly visualised cellular parts for genes with an upregulated expression upon both flavonoids and their 1:1 treatment mixtures in cellular compartment (CC) analysis (Figure 9A). The vacuolar (GO:0005773) and lysosomal (GO:0005764) compartments were mostly enriched upon the addition of 60 and 120 μM G, while the mitochondrial matrix (GO:0005759) and microtubule-organizing centre (GO:0005815) were enriched upon treatment with 60 and 120 μM K, respectively. For 60 and 120 μM G and K 1:1 mixtures, both cellular compartments were mostly enriched. Protein products of negatively regulated transcripts were involved in the nucleosome (GO:0000786), cytosol (GO:0005829), and extracellular region (GO:0044421) in 60 and 120 μM G. The extracellular region (GO:0044421), focal adhesion (GO:0005925), and nucleosome (GO: 0000786) were mostly enriched cell parts in GO terms upon treatment with K alone and with the 1:1 ratio of the G and K mixture at both concentrations. According to analysis performed for the biological process (BP), the products of genes upregulated and downregulated by flavonoids were mainly included in regulatory processes in the cell (Figure 9B), and the contribution of genes with downregulated expression was predominant, especially in G-treated samples. Positively regulated genes were mostly represented for flavonoids at both concentrations tested in the following: vacuole organization (GO:0007033), regulation of macroautophagy (GO:0016241), and vacuolar transport (GO:0007034) for G; glutathione metabolic process (GO:0006749), cofactor metabolic process (GO:0051186), and microtubule-based protein transport (GO:0099118) for K; and cellular response to DNA damage stimulus (GO:0006974), glutathione metabolic process (GO:0006749), and vacuole organization (GO:0007033) for the 1:1 flavonoid mixture. Negatively regulated transcripts were principally presented in GO terms related to the mitotic cell cycle process (GO:1903047), nucleosome assembly (GO:0006334), and chromosome organization (GO:0051276) upon treatment with 60 and 120 μM G; regulation of developmental process (GO:0050793), cell adhesion (GO:0007155), and extracellular matrix organization (GO:0030198) upon exposure to 60 and 120 μM K; and stimulation to protein heterotetramerization (GO:0051290), regulation of cell proliferation (GO:0042127), and regulation of cellular component movement (GO:0051270) upon addition of 60 and 120 μM of the 1:1 flavonoid mixture. The number of BPs downregulated in GO terms was minor for G-treated samples when compared to that for K alone and the flavonoid mixture, but the enrichment was much more potent. 

### 2.10. Genes Expressing Proteins Localised on the Lysosomal Membrane and Glycosidases Are Positively Regulated by Flavonoids

**Figure 10:**
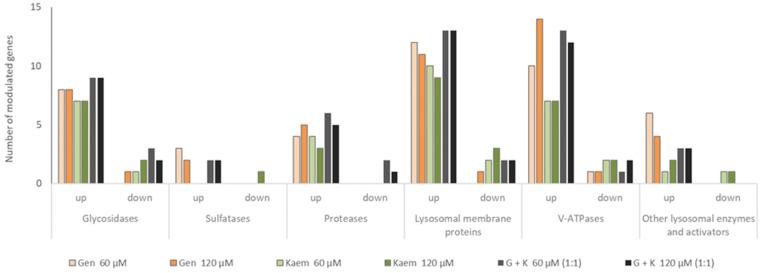
Microarray analysis of murine MPSI fibroblast genes coding for lysosomal proteins with a modulated expression upon treatment with genistein (Gen), kaempferol (Kaem), and genistein and kaempferol 1:1 mixture (G + K) at 60 and 120 μM final concentrations.

Considering modulation of expression of genes coding for GAG degradation, analysis of whole lysosomal protein-coding genes was performed. Out of 135 genes, 54 and 51 were modulated for the 60 and 120 μM of the 1:1 mixture of G and K, respectively. In the case of G, the number of regulated transcripts reached 31 and 33 for 60 and 120 μM and 26 and 28 for 60 and 120 μM K, respectively. Taking into account groups of enzymes, the most significant impacts of G, K, and their 1:1 mixture in both tested concentrations was observed for lysosomal membrane protein transcripts (*Abca2, Cd68, Cln3, Cln5, Ctns, Hgsnat, Lamp2, Laptm4b, Mcoln1, Mfsd8, Npc1, Npc2, Scarb2, Slc17a5, Slc11a2,* and *Sort1*), V-ATPases (*ATP6V0A3, Atp6v0a1, Atp6v0a2, Atp6v0b, Atp6v0e, Atp6v0e2, Atp6v1a, Atp6v1b2, Atp6v1d, Atp6v1e1, Atp6v1e2, Atp6v1f, Atp6v1g1, Atp6v1g2,* and *Atp6v1h*), and glycosidases (*Gla, Glb1l, Fuca1, Hexb, Hexdc, Hyal1, Manba, Man2b1, Naglu,* and *Neu1*), respectively (Figure 10). It is noteworthy that the analysed genes were predominantly upregulated for all conditions tested (Table S3). 

### 2.11. Flavonoids Have Significant Impacts on the Expression of Genes Involved in Lysosomal Metabolism, Galectins, and Transcription Factors

In addition to microarray and Gene Ontology analyses, real-time qRT-PCR was performed for genes with both modulated expression and prominent roles in MPS I. Among them, expression of lysosomal metabolism-protein transcripts, galectins, melanocyte inducing transcription factor (MITF) family, and transforming growth factor beta (TGFβ) was determined (Table 3). 

Analysis of lysosomal protein-coding genes selected from diverse groups of enzymes displayed positive regulation of expression of all transcripts of both glycosidases (*Fuca1, Gla, Man2b1,* and *Neu1*), proteases (*Ctsk*), lysosomal membrane proteins (*Ctns, Mcoln1, Npc1, Npc2, Slc17a5,* and *Sort1*), and other lysosomal enzymes and activators (*Aga, Gm2a,* and *Ppt1*) in almost all tested conditions (Table 3). 

Our previous results demonstrating modulation of TFEB and other factors belonging to the microphthalmia family of transcription factors (MiT) expression by flavonoids prompted us to investigate whether treatment with the flavonoid mixture may effectively affect the expression of the CLEAR-gene network, activating lysosomal biogenesis and commanded by TFEB. Unexpectedly, there was no activity modulation of the murine EB transcription factor homologue, *Tcfeb*, in all tested conditions. Analyses conducted for other transcription factors from the MiT family also revealed no changes in the expression level of *Tcfec*. Negative modulation was seen for *Mitf* after treatment with 120 µM G but in contrary positive regulation after 120 µM K and 120 µM 1:1 mixture of G + K. In addition, upregulated expression of *Tcfe3* was observed upon treatment with 120 µM K and 120 µM 1:1 mixture of G + K (Table 4). 

The study also revealed significant decreases in the activity levels of genes coding for murine galectins, namely galectin 3 (*Lgals3*), 9 (*Lgals9*), and 1 (*Lgals1*), respectively, upon treatment with flavonoids. However, upregulation of the galectin 8 gene (*Lagls8*) was noted. Proteins belonging to the galectin family are well-known modulators of the inflammatory response, with prevalent roles for galectins 1, 3, and 9 (Table 4). 

TGFβ is a cytokine with an established role in GAG biosynthesis. Modulation of TGFβ and its receptor transcript isoforms was also observed with upregulation of *Tgfb3, Tgfbr1*, and *Tgfbr3* and downregulation of *Tgfb1, Tgfb1*, and *Tgfb2*. These modulations were noted for almost all tested conditions (Table 4).

## 3. Discussion

Multi-compound combinations are often prescribed in the practice of clinical medicine and as medical use of nutrition for special purposes. The main motivations for these combinations are that most diseases contain multiple related targets and an appropriate combination can maximise benefits while minimising adverse reactions [15]. In traditional Chinese medicine, combinations of various drugs derived from animals, plants, and minerals are frequently used as both medicine and food or as a fixed prescription. The activity of many medicinal plants results from the interaction of several constituents, which may cooperatively act in an additive or synergistic manner. It has been repeatedly observed that extracts of medicinal plants reveal better activities than their isolated single compounds at comparable equivalent concentrations of the active components. This phenomenon is attributed to the absence of interacting substances present in crude extracts. In pharmacokinetic synergy, substances with little or no bioactivity may assist the main active principle to reach the disease target by several mechanisms (e.g., improving bioavailability or decreasing metabolism and excretion). 

Among various phytochemicals, the most attention is paid to flavonoids due to their considerable biological activity and potential medicinal benefits [2]. Traditional and modern medicine has always taken advantage of the combined use of several active agents to treat different diseases. Many of the most effective phytomedications on the pharmaceutical market most often occur as whole plant extracts. Therefore, carefully choosing the proper extract is necessary for its therapeutic efficacy [16]. Moreover, a better understanding of the molecular basis of disease and the metabolic pathways that are impaired in a condition provides the opportunity to develop combination therapies where several active agents interact simultaneously. Such a therapeutic approach exploits the chance for better efficacy, decreased toxicity, and reduced development of drug resistance. Therefore, a wide spectrum of biological activities, which flavonoids have, gives a huge perspective of their use as natural drugs in the treatment of various diseases, including some genetic disorders [16,17]. For this purpose, it is highly important to understand and determine the type and degree of interactions between flavonoids in a mixture.

One of a group of genetic diseases in which the use of flavonoids has a therapeutic effect is mucopolysaccharidoses (MPS). In MPS, the metabolism of GAGs is altered due to a complete lack of or minor residual activity of specific lysosomal acid hydrolases. This results in the intra- and extracellular accumulation of improperly degraded GAGs, leading to progressive visceral organ storage, neurodegeneration, neuroinflammation, and premature death of affected patients. The development of therapeutic strategies for MPS is limited because the blood–brain barrier (BBB) is highly impermeable to large therapeutic molecules, such as enzymes used in enzyme replacement therapy (ERT); thus, the symptoms of neurological disease are difficult to ameliorate. Alternatively, low molecular weight molecules used in SRT could potentially readily cross the BBB and benefit the treatment of disease by reducing the intracellular production of GAGs to reduce pathological storage and slow down the disease progression in affected patients. 

Genistein (G), a soy isoflavone, has already been described as a potent inhibitor of GAG synthesis in fibroblasts [8]. It also can modulate lysosomal biogenesis and the GAG degradation pathway [18,19]. Furthermore, short-term administration of genistein significantly reduced GAG content and lysosomal compartment size in the liver of MPS IIIB mice [10] and the liver, spleen, kidney, and heart of MPS II mice [9]. This isoflavone also corrected the neuropathology and behaviour of MPS IIIB mice following long-term treatment [11]. Administration of 160 mg/kg/day genistein over 9 months resulted in a significant reduction of brain GAG and GM2 ganglioside storage, a decrease in neuroinflammatory parameters, and normalisation of the behaviour of animals [11]. Soy isoflavone extracts rich in genistein effectively decreased urinary GAG levels and the improved cognitive function of MPS III patients [17,20,21] or had a significant impact on the range of joint motion in MPS II patients [22]. However, other studies have reported only minor [23] or no effects [24] following treatment of MPS patients with genistein. 

Other flavonoids, including kaempferol, daidzein, formononetin, glycitein, or mixtures of them, reduce GAG synthesis and storage in cultured fibroblasts of MPS types IIIA, IIIB, and VII [12,13]. Considering the above studies, flavonoids were suggested as potential active agents in SRTs for MPS. 

Recently, a *Medicago sativa* extract containing daidzein, formononetin, genistein, and glycitein was also shown to significantly reduce the GAG amount in MPS IIIB cells. However, the extract activity was lower compared to treatment with pure genistein [25]. The differences in efficiency can be caused by interactions between all biologically active components contained in the extract. Understanding the nature of the interaction between the individual components in the tested formulation is crucial for the correct evaluation of its effectiveness. Combination therapies exploit the chance for better efficacy with decreased toxicity and have become a promising treatment approach for many medical conditions. As genistein significantly reduces cell proliferation [26], we propose to decrease the concentration of this isoflavone by combination with other flavonoids in a mixture. Significant dose reductions obtained for genistein used in combination with kaempferol suggest that this approach may considerably diminish the unwanted effects of genistein. Gene expression analysis performed for flavonoids alone and 1:1 combinations demonstrated that mixtures of genistein and kaempferol at a 1:1 ratio were most potent, regulating expression of 52% of GAG pathway metabolism genes, suggesting that combining flavonoids may serve as a proper therapeutic solution (Figure 7). Differences in effect resulting from tested concentrations were not prominent for this and also for whole-genome analysis. Regarding modulation of genes coding specifically for lysosomal proteins, we have noticed significant upregulation of expression of numerous transcripts of lysosomal membrane proteins (Figure 10). They are central for the biogenesis of this compartment and are considered attractive targets to modulate the lysosomal machinery in cases where impaired lysosomal degradation leads to cellular pathologies, such as MPS and other LSDs. Elevated lysosomal biogenesis was observed upon all conditions and was related to TFEB, a lysosomal biogenesis factor, and nuclear translocation. This, in turn, may be due to the upregulation of the galectin 8 coding gene (Table 4). Galectin 8 is necessary to stop mTORC1 signalling in response to lysosomal damage. The interaction of galectin 8 with Rag GTPases and Ragulator results in loss of mTORC1 lysosomal localisation and activity, which prompts the shuttling of TFEB from the cytoplasm to the nucleus [27]. Moreover, expression of V-ATPases and glycosidases, playing key roles in autophagy and protein degradation, was also upregulated, indicating intensified degradation of lysosomal deposits first and secondary storage material in LSDs. Gene Ontology analysis revealed vacuolar and lysosomal compartments and vacuole organization as mostly enriched positively modulated terms upon treatment with flavonoid mixtures. It is worth mentioning that terms assigned to the cell cycle and proliferation were not as enriched among genes negatively regulated upon treatment with the genistein and kaempferol mixture, as it was for genistein alone (Figure 9). This isoflavone has an apparent impact on the G1/S, G2/M, and M protein-coding gene phases of the cell cycle (Figure 8).

**Figure 11:**
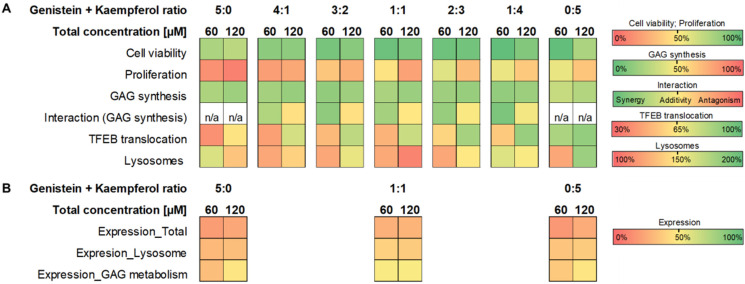
Summary of the effects of flavonoid combinations on cell viability, proliferation, GAG synthesis, and type of interactions (**A**), as well as gene expression profile, TFEB translocation, and number of lysosomes (**B**) between flavonoids in two-component mixtures. Fields shaded in green correspond to positive effects (i.e., cell viability greater than 60%, cell proliferation greater than 60%, and GAG synthesis level lower than 40% of control cells), in yellow correspond to intermediate effects (around 50%), and in red correspond to negative effects (values not meeting positive effects criteria). The type of interaction between flavonoids regarding the GAG synthesis process is assigned as synergy (green, CI < 0.90), additivity (yellow, 0.90 < CI < 1.10), or antagonism (red, CI > 1.10).

According to our results, the interactions observed between flavonoids, such as genistein and kaempferol applied in mixtures, include antagonism, synergy, or additivity regarding the process of GAG synthesis. The type of interactions strongly depends on the mixture composition, concentration, and component ratios used (see the summary in Figure 11A). 

Although genistein and other flavonoids are non-toxic in many in vitro experiments [12,13], there is a huge inconsistency in the results on the safety of using genistein in animal [28] or human models [24]. Our present study indicated that for different flavonoid mixtures, increasing amounts of kaempferol in place of genistein resulted in less prominent proliferation inhibition (summarised in Figure 11A). Moreover, at the highest total concentration of 120 µM for all tested flavonoid mixtures, we observed a negative effect on cell viability and proliferation (summarised in Figure 11A), which was supported by Gene Ontology analysis (Figure 11B). Considering that increasing the total concentration of flavonoids results in antagonism in the modulation of GAG synthesis between both components used in a mixture, regardless of the drug ratio used, we propose that the best flavonoid combinations to be used are the mixtures of genistein and kaempferol, both at 2:3 and 1:4 ratios in 60 µM total concentration (Figure 11A). The effect on cell proliferation and viability is acceptable, and the synergy in GAG synthesis modulation is observed. Since natural flavonoid extracts are characterised by different compositions regarding both components and ratios, one should be very cautious while using such plant extracts or mixtures of natural compounds, not only in the case of SRT for mucopolysaccharidosis, but also in cases of using flavonoids as dietary supplements to provide many health benefits. 

The composition and content of specific components in commercially available isoflavone extracts are not always fully determined [16,29]; thus, inconsistency between results obtained from clinical studies with MPS patients using pure genistein (e.g., recently completed GENiSIS2013 clinical trial, EudraCT Number 2013-001479-18) or soy isoflavone extracts are possible [17,20,21,23]. As we have demonstrated for genistein and kaempferol, the efficiency and potency to modulate different biological processes depend strongly on the composition and ratio of active compounds in a mixture. We estimated that the concentration of genistein, in the genistein–kaempferol mixture, required to achieve effective reduction of GAG synthesis could be substantially decreased but only at the specific ratios of the components (see the summary in Figure 11A). The analysis of gene expression revealed the advantage of a combination of flavonoids over the impact of single flavonoids (Figure 11B), but according to our results, the content of flavonoids in a mixture, as well as their concentration, should always be very precisely selected and examined regarding the process of interest, because interactions may vary between different mixture components or their ratios. 

Our results provide data that two-component mixtures consisting of genistein and kaempferol at certain ratios are optimal for reduction of GAG synthesis, and these specified flavonoid compositions could be used as active compounds in SRT for mucopolysaccharidosis. Further in vivo studies are necessary to determine whether these compositions are effective, and if so, to what extent in the correction of the disease phenotype.

## 4. Materials and Methods

### 4.1. Culture Media, Culture Conditions, and Reagents

Cells were cultured in Dulbecco’s modified Eagle’s medium (DMEM) supplemented with 10% foetal bovine serum (FBS) and 1% antibiotic/antimycotic solution (Gibco™, all purchased from Thermo Fisher Scientific, Bleiswijk, The Netherlands), at 37 °C in a humidified atmosphere containing 5% carbon dioxide (CO_2_). Kaempferol (3,5,7-trihydroxy-2-(4-hydroxyphenyl)chromen-4-one) was purchased from AK Scientific, Inc. (Union City, CA, USA). Genistein (5,7-dihydroxy-3-(4-hydroxyphenyl)chromen-4-one) was synthesised by the Pharmaceutical Research Institute (Warsaw, Poland). Stock solutions of flavonoids were dissolved in dimethyl sulfoxide (DMSO) (Sigma-Aldrich, Darmstadt, Germany) and were stored at −20 °C in the dark. Working solutions of flavonoids were freshly prepared in a culture medium at appropriate concentrations depending on the experiment. Mixtures of genistein and kaempferol were prepared in DMEM culture medium for total concentrations of 30, 60, and 120 µM and ratios of 5:0, 4:1, 3:2, 2:3, 1:4, 0:5, and 1:1 of indicated compounds. For experimental procedures, cells were plated to a confluence of approximately 80%. After overnight incubation, the culture medium was replaced with a fresh medium either supplemented with appropriate amounts of tested flavonoids or the one containing DMSO at a final concentration of 0.05% or 0.1% for a single flavonoid or flavonoid mixture, respectively. 

Glucosamine, D-[1-3H] hydrochloride (3H-GlcN) was purchased from Hartmann Analytic GmbH (Braunschweig, Germany), and Invitrogen™ Quant-iT™ PicoGreen^®^ dsDNA reagent was purchased from LifeTechnologies (Bleiswijk, The Netherlands).

### 4.2. Derivation of Primary Skin Fibroblasts from Mouse Tails 

Animal maintenance and all procedures were ethically approved and carried out following the Polish local ethical committee regulations. Mice at the age of 6–8 weeks were sacrificed by cervical dislocation, and their tails were immediately cut off and placed in phosphate-buffered saline (PBS) (Gibco™, Thermo Fisher Scientific, Bleiswijk, The Netherlands) with 2% antibiotic–antimycotic solution. Under sterile conditions, skin from the tail was removed and cut into 2–3 mm squares. Five to six skin pieces were placed in the centre area of a 6-well plate and allowed to air dry for 5 min. After that time, a drop of 37 °C DMEM supplemented with 10% FBS and 1% antibiotic–antimycotic solution was added to each tissue fragment, followed by an extra 1–2 mL of medium replenished with caution to not detach the skin from the bottom of the dish, and placed in a humidified 37 °C, 5% CO_2_ incubator. Skin explants were cultured for 10 days, with fresh medium added every second day. After that time, when fibroblasts effectively migrated from the tissue, skin fragments were removed, and a fresh complete medium was added to each well. When cultures reached around 80% confluency, cells were washed with DMEM without FBS and trypsinised with 1× trypsin-EDTA solution. The cell suspension was transferred to a new culture dish and considered as passage one.

### 4.3. Cytotoxicity Assay

Cytotoxicity was assessed using the CellTox™ Green Express Cytotoxicity Assay (Promega, Walldorf, Germany) as a measure of compromised cell membrane integrity resulting from cell death. In brief, cells were seeded at 10⁴ cells per well of a black 96-well culture plate. Following overnight incubation at 37 °C, the culture medium supplemented with 1× CellTox™ Green Dye and appropriate concentrations of a single flavonoid or their mixtures were added to wells. After 4, 24, 48, and 72 h of incubation at 37 °C, fluorescence was measured at 512/532 nm, and cytotoxicity was calculated relative to positive cytotoxicity controls (100% dead cells) run within the experiment.

### 4.4. Cell Viability and Proliferation Assay

Cells were seeded at 5 × 10^3^ cells per well (cell viability assay) or 4 × 10^3^ cells per well (proliferation assay) of 96-well culture plates. Following overnight incubation at 37 °C, cells were exposed to different concentrations of single flavonoids or their mixtures in a DMEM culture medium. Cell viability was assessed after 4 and 24 h exposure to tested compounds using 3-(4,5-dimethylthiazol-2-yl)-2,5-diphenyltetrazolium bromide (Sigma-Aldrich, Darmstadt, Germany) dissolved at a concentration of 1 mg/mL in RPMI-1640 medium without phenol red (MTT dye solution). Following a 2 h incubation of cells with MTT dye solution, purple formazan was dissolved in DMSO (Sigma-Aldrich, Darmstadt, Germany), and absorbance was read at 570 nm. Cell proliferation was assessed by the measurement of bromodeoxyuridine (BrdU) incorporation after a 45 h exposure of cells to tested compounds using the Cell Proliferation ELISA, BrdU (colourimetric) Assay Kit (Roche Applied Science, Indianapolis, IN, USA) according to the standard protocol.

### 4.5. Measurement of GAG Synthesis Kinetics

The kinetics of GAG synthesis in cell cultures exposed to different concentrations of flavonoids or their mixtures were assessed by measuring the incorporation of 3H-GlcN as described by Moskot et al. [19]. In brief, 2 × 10^4^ cells were plated per well in a 48-well plate. Following overnight incubation to allow cells to adhere, they were exposed to appropriate concentrations of flavonoids or flavonoid mixtures for 24 h. Next, cells were labelled with 10 µCi/mL of 3H-GlcN in DMEM without glucose and pyruvate supplemented with 10% FBS and a single flavonoid, their mixtures (G + K, G + B, and K + B), or DMSO alone (control cultures) for an additional 24 h. Following their labelling, cells were washed with PBS and digested with 0.5% papain. The level of 3H-GlcN incorporation into GAGs in papain digested samples was measured using a MicroBeta2 scintillation counter (PerkinElmer, Waltham, MA, USA), and the DNA concentration was quantified with Quant-iT™ PicoGreen^®^dsDNA reagent. The incorporation of 3H-GlcN was standardised against the DNA amount (cpm/ng of DNA). The efficiency of GAG synthesis in flavonoid-treated cultures was expressed as relative to control cultures (treated with DMSO alone).

### 4.6. RNA Extraction

Total RNA was extracted from cells using the High Pure RNA Isolation Kit (Roche Applied Science, Indianapolis, IN, USA) following the manufacturer’s instructions. The quality and quantity of each RNA sample were evaluated using the RNA 6000 Nano Assay on the Agilent 2100 Bioanalyzer (Agilent Technologies Inc., Santa Clara, CA, USA).

### 4.7. Microarray Assays for mRNA Analysis

Whole-genome microarray analysis of three biological replicates (*n*) was performed using the Clariom S mouse assay (Affymetrix, Thermo Fisher Scientific, Waltham, MA, USA) (>22,100 genes, >150,300 transcripts, and >221,900 probes). The assay performance, data extraction, and statistical analysis were performed as previously described [18]. Gene Ontology analysis and data visualisation were performed using the web tools GOrilla (http://cbl-gorilla.cs.technion.ac.il/, (accessed on 2 December 2020)) [30] and REViGO (http://revigo.irb.hr/, (accessed on 10 December 2020), restricting the output to biological process and cell compartment. Gene Set Enrichment Analysis (GSEA) was performed on the upregulated and downregulated gene lists, separately as previously described [31,32]. A nominal *p*-value < 0.01 and a false discovery rate (FDR) < 0.25 were used to assess the significance of the enrichment scores. The Kyoto Encyclopedia of Genes and Genomes (KEGG) (GenomeNet, http://www.genome.jp/kegg/ (accessed on 10 January 2021) database was used for the selection of genes related to specifically selected pathways.

### 4.8. Quantitative Real-Time RT-PCR Array for mRNA Analysis

Total RNA was retrotranscribed with the Transcriptor First Strand cDNA Synthesis Kit (Roche Applied Science, Indianapolis, IN, USA). Real-time quantitative RT-PCR was carried out with the RealTime ready Custom Panel (cat no. 05532914001, config. no. 100132522, Roche Applied Science, Indianapolis, IN, USA) and the LightCycler^®^ 480 Probes Master (Roche Applied Science, Indianapolis, IN, USA) using the Light Cycler 480 II detection system (Roche). Expression values were normalized against the *B2m*, *Gapdh*, and *Hmbs* control genes. For both microarray and real-time qRT-PCR analyses, a fold change (FC) greater or equal to 1.3 and below 0.7 was considered as a relevant criterion for genes being significantly differentially expressed RNA.

### 4.9. Fluorescence Assays

TFEB-FLAG HeLa cells were seeded on 96-well plates, incubated for 24 h, and treated with genistein, kaempferol, and mixtures of both compounds ranging from 1 to 150 µM, as well as 0.3 µM Torin 1 (Biomarin Pharmaceutical, London, UK), in an FBS-free medium. The proportions of flavonoid mixtures were established based on isobologram analysis. After 3 h at 37 °C, cells were washed, fixed, and stained with 4′,6-diamidino-2-phenylindole (DAPI) (Sigma-Aldrich, Darmstadt, Germany). For the acquisition of the images, 10 pictures per well of the 96-well plate were taken by using confocal automated microscopy (Opera High Content Screening system; Perkin-Elmer) with 40× magnification. A dedicated script calculating the ratio value resulting from the average intensity of nuclear TFEB-GFP fluorescence divided by the average of the cytosolic intensity of TFEB-GFP fluorescence was developed to perform the analysis of TFEB localisation on the different images (Acapella software, Perkin-Elmer, Waltham, MA, USA). To visualise the acidophilic compartments of the cell, we used Lysotracker Red DN-99 (Molecular Probes, Life Technologies, Bleiswijk, The Netherlands). ARPE-19 cells seeded on the 96-well plate were incubated with 75 nm Lysotracker Red DN-99 for 1 h at 37 °C, rinsed with PBS and fixed with 4% paraformaldehyde in PBS for 15 min at RT. Counterstaining was done with DAPI. Images were taken using confocal automated microscopy (Opera High Content Screening system; Perkin-Elmer, Waltham, MA, USA) with 60× magnification.

### 4.10. Data Analysis and Statistics

The effective concentration of the tested flavonoids resulting in a 50% reduction of cell response (EC50 values) was determined from sigmoidal dose–response curves using four-parameter logistic equations. To characterise the interactions between flavonoids, an isobolographic analysis was performed using CompuSyn 1.0 software (ComboSyn, Inc., Paramus, NJ, USA) [33] to determine the combination index (CI) and dose reduction index (DRI). Drug interactions were assessed with the following values: CI < 1 for synergy, CI = 1 for additive, and CI > 1 for antagonism. Favourable dose reduction was representative of a DRI > 1. To test the effects of flavonoid mixtures on cell viability, proliferation, and GAG synthesis, one-way ANOVA was performed with Tukey’s post hoc test. Significance was declared at *p* < 0.05. 

## 5. Conclusions

Mixtures of genistein and kaempferol are effective modulators of glycosaminoglycan metabolism and lysosomal biogenesis—important processes in the cellular turnover of these macromolecules. With the simultaneous use of both flavonoids, it is possible to obtain synergy of their action and reduce the concentration of components, while maintaining the cellular response at a similar level as that observed for these compounds when used individually, but at higher concentrations. Hence, mixtures of flavonoids, rather than single components, seem to be promising potential agents in the treatment of mucopolysaccharidosis.

## Figures and Tables

**Table 1:** Combination index (CI) values and the type of interaction between genistein and kaempferol as determined regarding their impact on GAG synthesis. The type of interaction in a combination was quantified according to CI values with the following categories: synergism (0.30–0.70), moderate synergism (0.70–0.85), nearly additive (0.90–1.10), slight antagonism (1.10–1.20), moderate antagonism (1.20–1.45), and antagonism (1.45–3.3). Dose reduction index (DRI) values were calculated at experimental points (effect level = experimental levels of GAG synthesis inhibition). DRI indicates the fold of dose reduction for each drug in a synergistic combination at a given effect level when compared to the dose of each drug used alone. DRI = 1 indicates no dose reduction, DRI > 1 indicates favourable dose reduction, and DRI < 1 indicates unfavourable dose reduction. The CI and DRI values were estimated using CompuSyn 1.0 software.

Concentration	Ratio	Genistein + Kaempferol		DRI	
		CI	Interaction	Genistein	Kaempferol
30 µM	4:1	0.71	Moderate synergism	1.86	5.74
	3:2	0.60	Synergism	2.90	3.92
	2:3	0.64	Synergism	4.24	2.47
	1:4	1.16	Slight antagonism	6.21	1.00
60 µM	4:1	0.65	Synergism	1.80	10.69
	3:2	0.50	Synergism	2.76	7.05
	2:3	0.54	Synergism	3.68	3.71
	1:4	0.42	Synergism	8.08	3.35
120 µM	4:1	1.17	Slight antagonism	0.99	6.37
	3:2	1.17	Slight antagonism	1.23	2.78
	2:3	0.98	Nearly additive	1.97	2.12
	1:4	0.95	Nearly additive	3.76	1.45

**Table 2:** Microarray and real-time qRT-PCR expression profile of murine MPS I fibroblast genes coding for GAG metabolism with expression modulated upon 24 h of flavonoid treatment. Genes with upregulated expression are marked in red, while those with downregulated expression are marked in blue.

	Gene Symbol	Genistein 120 µM				Kaempferol 120 µM				Genistein + Kaempferol 120 µM (1:1)			
		Microarray	qRT-PCR			Microarray	qRT-PCR			Microarray	qRT-PCR		
			vs. B2m	vs. Gapdh	vs. Hmbs		vs. B2m	vs. Gapdh	vs. Hmbs		vs. B2m	vs. Gapdh	vs. Hmbs
		FC ± SD	FC ± SD	FC ± SD	FC ± SD	FC ± SD	FC ± SD	FC ± SD	FC ± SD	FC ± SD	FC ± SD	FC ± SD	FC ± SD
GAG synthesis	*B3gnt2*	1.0 ± 0.0	0.9 ± 0.1	1.0 ± 0.2	1.1 ± 0.0	0.4 ± 0.0	0.4 ± 0.1	0.5 ± 0.0	0.4 ± 0.0	0.5 ± 0.0	0.5 ± 0.0	0.4 ± 0.1	0.5 ± 0.1
	*B4galt1*	0.8 ± 0.0	0.8 ± 0.0	0.7 ± 0.1	0.7 ± 0.0	0.7 ± 0.0	0.6 ± 0.1	0.7 ± 0.0	0.7 ± 0.2	0.6 ± 0.1	0.6 ± 0.0	0.7 ± 0.0	0.6 ± 0.1
	*B4galt4*	2.2 ± 0.4	2.3 ± 0.3	2.0 ± 0.1	1.9 ± 0.2	3.3 ± 0.0	3.1 ± 0.1	3.3 ± 0.2	3.4 ± 0.1	2.6 ± 0.1	2.8 ± 0.3	2.5 ± 0.1	2.6 ± 0.2
	*Csgalnact1*	1.8 ± 0.0	1.8 ± 0.1	1.9 ± 0.2	2.1 ± 0.0	3.0 ± 0.5	2.8 ± 0.3	3.1 ± 0.2	3.0 ± 0.3	3.8 ± 0.2	3.8 ± 0.3	3.4 ± 0.1	3.5 ± 0.2
	*Chst2*	2.0 ± 0.2	2.0 ± 0.2	1.9 ± 0.3	2.2 ± 0.1	2.5 ± 0.3	2.4 ± 0.2	2.5 ± 0.3	2.5 ± 0.1	3.1 ± 0.0	3.1 ± 0.2	3.3 ± 0.0	3.3 ± 0.1
	*Chsy1*	1.3 ± 0.1	1.2 ± 0.0	1.3 ± 0.2	1.3 ± 0.0	1.6 ± 0.3	1.6 ± 0.1	1.7 ± 0.3	1.4 ± 0.0	1.7 ± 0.0	1.7 ± 0.1	1.6 ± 0.3	1.7 ± 0.1
	*Ext1*	0.7 ± 0.0	0.7 ± 0.2	0.7 ± 0.1	0.6 ± 0.0	0.6 ± 0.0	0.6 ± 0.0	0.5 ± 0.0	0.6 ± 0.1	0.6 ± 0.0	0.6 ± 0.1	0.6 ± 0.0	0.7 ± 0.1
	*Extl3*	1.6 ± 0.0	1.5 ± 0.1	1.6 ± 0.1	1.6 ± 0.0	1.6 ± 0.1	1.6 ± 0.0	1.5 ± 0.1	1.5 ± 0.2	1.4 ± 0.1	1.4 ± 0.2	1.3 ± 0.0	1.3 ± 0.1
	*Dse*	0.7 ± 0.0	0.6 ± 0.1	0.7 ± 0.0	0.7 ± 0.1	0.5 ± 0.0	0.5 ± 0.0	0.4 ± 0.1	0.5 ± 0.1	0.5 ± 0.0	0.6 ± 0.0	0.5 ± 0.1	0.6 ± 0.1
	*Ndst2*	0.7 ± 0.1	0.5 ± 0.0	0.6 ± 0.1	0.7 ± 0.0	0.6 ± 0.0	0.6 ± 0.0	0.7 ± 0.2	0.6 ± 0.1	0.6 ± 0.0	0.6 ± 0.1	0.6 ± 0.0	0.5 ± 0.1
GAG degradation	*Hexb*	1.7 ± 0.1	1.7 ± 0.1	1.5 ± 0.2	1.7 ± 0.1	1.5 ± 0.1	1.4 ± 0.0	1.7 ± 0.2	1.5 ± 0.0	1.8 ± 0.0	1.7 ± 0.0	1.8 ± 0.1	1.8 ± 0.0
	*Hgsnat*	1.8 ± 0.1	1.3 ± 0.2	1.6 ± 0.1	1.3 ± 0.2	1.5 ± 0.1	1.5 ± 0.2	1.3 ± 0.1	1.5 ± 0.0	1.4 ± 0.1	1.3 ± 0.1	1.4 ± 0.0	1.5 ± 0.2
	*Hyal1*	4.4 ± 0.1	4.1 ± 0.3	4.5 ± 0.4	3.9 ± 0.3	2.7 ± 0.3	2.3 ± 0.1	2.6 ± 0.1	2.7 ± 0.2	4.6 ± 0.1	4.4 ± 0.2	4.7 ± 0.1	5.0 ± 0.2
	*Naglu*	1.4 ± 0.1	1.6 ± 0.1	1.5 ± 0.2	1.8 ± 0.2	1.5 ± 0.1	1.5 ± 0.0	1.4 ± 0.1	1.6 ± 0.2	1.5 ± 0.2	1.6 ± 0.2	1.4 ± 0.0	1.4 ± 0.1
	*Sgsh*	1.4 ± 0.2	1.3 ± 0.2	1.4 ± 0.2	1.3 ± 0.2	1.0 ± 0.2	1.0 ± 0.0	1.1 ± 0.2	1.0 ± 0.1	1.4 ± 0.0	1.4 ± 0.1	1.3 ± 0.2	1.4 ± 0.0

**Table 3:** Microarray and real-time qRT-PCR expression profile of murine MPS I fibroblast genes coding for lysosomal proteins with expression modulated upon flavonoid treatment. Genes with upregulated expression are marked in red.

	Genistein 120 µM, 24 h				Kaempferol 120 µM, 24 h				Genistein + Kaempferol 120 µM (1:1)			
Gene Symbol	Microarray	qRT-PCR			Microarray	qRT-PCR			Microarray	qRT-PCR		
		vs. B2m	vs. Gapdh	vs. Hmbs		vs. B2m	vs. Gapdh	vs. Hmbs		vs. B2m	vs. Gapdh	vs. Hmbs
	FC ± SD	FC ± SD	FC ± SD	FC ± SD	FC ± SD	FC ± SD	FC ± SD	FC ± SD	FC ± SD	FC ± SD	FC ± SD	FC ± SD
*Aga*	2.4 ± 0.2	2.1 ± 0.0	2.3 ± 0.2	2.1 ± 0.0	2.2 ± 0.2	2.0 ± 0.2	1.8 ± 0.1	2.2 ± 0.1	2.2 ± 0.0	2.2 ± 0.2	1.9 ± 0.0	2.3 ± 0.1
*Ctns*	1.7 ± 0.2	1.5 ± 0.2	1.9 ± 0.1	1.5 ± 0.2	2.3 ± 0.1	2.3 ± 0.1	2.1 ± 0.2	1.9 ± 0.1	1.8 ± 0.0	1.7 ± 0.1	1.9 ± 0.2	1.8 ± 0.0
*Ctsk*	0.7 ± 0.0	0.8 ± 0.1	0.7 ± 0.0	0.8 ± 0.1	1.7 ± 0.1	1.6 ± 0.1	1.7 ± 0.3	1.9 ± 0.2	1.3 ± 0.1	1.3 ± 0.2	1.2 ± 0.1	1.3 ± 0.1
*Fuca1*	2.5 ± 0.0	1.9 ± 0.1	2.2 ± 0.2	1.9 ± 0.1	1.6 ± 0.2	1.5 ± 0.1	1.6 ± 0.1	1.8 ± 0.2	2.1 ± 0.0	2.0 ± 0.2	2.1 ± 0.0	2.0 ± 0.1
*Gla*	1.7 ± 0.0	1.9 ± 0.0	1.7 ± 0.0	1.9 ± 0.0	2.1 ± 0.4	1.9 ± 0.2	2.1 ± 0.2	2.3 ± 0.3	1.8 ± 0.1	1.8 ± 0.0	1.8 ± 0.1	1.9 ± 0.1
*Gm2a*	1.2 ± 0.1	1.5 ± 0.1	1.3 ± 0.1	1.5 ± 0.1	1.1 ± 0.1	1.0 ± 0.0	1.3 ± 0.1	1.1 ± 0.1	1.5 ± 0.1	1.6 ± 0.1	1.5 ± 0.0	1.5 ± 0.1
*Man2b1*	1.9 ± 0.4	1.7 ± 0.2	1.8 ± 0.2	1.6 ± 0.3	1.3 ± 0.1	1.3 ± 0.0	1.1 ± 0.1	1.4 ± 0.1	1.5 ± 0.1	1.5 ± 0.2	1.4 ± 0.1	1.6 ± 0.0
*Mcoln1*	2.8 ± 0.4	2.6 ± 0.2	3.1 ± 0.3	2.5 ± 0.3	2.7 ± 0.1	2.6 ± 0.2	2.5 ± 0.3	2.7 ± 0.0	2.2 ± 0.0	2.1 ± 0.0	2.4 ± 0.2	2.2 ± 0.1
*Neu1*	3.0 ± 0.7	3.3 ± 0.4	2.8 ± 0.5	3.3 ± 0.1	1.8 ± 0.1	1.6 ± 0.1	1.7 ± 0.0	1.8 ± 0.2	2.3 ± 0.2	2.1 ± 0.2	2.5 ± 0.1	2.3 ± 0.0
*Npc1*	1.7 ± 0.2	1.7 ± 0.0	1.5 ± 0.2	1.6 ± 0.1	2.0 ± 0.2	2.0 ± 0.3	2.2 ± 0.1	1.9 ± 0.2	1.4 ± 0.0	1.3 ± 0.1	1.4 ± 0.2	1.6 ± 0.0
*Npc2*	1.6 ± 0.2	1.5 ± 0.1	1.8 ± 0.2	1.9 ± 0.1	1.6 ± 0.3	1.5 ± 0.1	1.6 ± 0.1	1.8 ± 0.0	1.7 ± 0.0	1.6 ± 0.2	1.8 ± 0.1	1.7 ± 0.3
*Ppt1*	2.3 ± 0.4	2.6 ± 0.5	1.9 ± 0.1	2.6 ± 0.5	1.2 ± 0.2	1.1 ± 0.0	1.1 ± 0.1	1.2 ± 0.0	1.5 ± 0.1	1.6 ± 0.0	1.4 ± 0.1	1.5 ± 0.2
*Slc17a5*	1.9 ± 0.5	2.1 ± 0.3	1.7 ± 0.2	2.2 ± 0.2	2.4 ± 0.1	2.3 ± 0.2	2.4 ± 0.3	2.5 ± 0.1	2.0 ± 0.1	2.2 ± 0.2	1.9 ± 0.1	2.0 ± 0.0
*Sort1*	1.9 ± 0.1	1.8 ± 0.2	2.0 ± 0.1	1.8 ± 0.1	2.1 ±0.2	2.1 ±0.1	1.9 ± 0.3	2.3 ± 0.2	3.6 ± 0.5	3.4 ± 0.3	3.8 ± 0.4	3.2 ± 0.2

**Table 4:** Expression profile of murine MPS I fibroblast genes coding for galectins and transcriptional factors with expression modulated upon 24 h flavonoid treatment. Genes with upregulated expression are marked in red, while genes with downregulated expression are marked in blue.

	Genistein 120 µM				Kaempferol 120 µM				Genistein + Kaempferol 120 µM (1:1)			
Gene Symbol	Microarray	qRT-PCR			Microarray	qRT-PCR			Microarray	qRT-PCR		
		vs. B2m	vs. Gapdh	vs. Hmbs		vs. B2m	vs. Gapdh	vs. Hmbs		vs. B2m	vs. Gapdh	vs. Hmbs
	FC ± SD	FC ± SD	FC ± SD	FC ± SD	FC ± SD	FC ± SD	FC ± SD	FC ± SD	FC ± SD	FC ± SD	FC ± SD	FC ± SD
*Lgals1*	0.7 ± 0.0	0.7 ± 0.1	0.6 ± 0.0	0.6 ± 0.1	0.8 ± 0.0	0.8 ± 0.1	0.7 ± 0.0	0.7 ± 0.1	0.7 ± 0.1	0.7 ± 0.0	0.7 ± 0.1	0.8 ± 0.1
*Lgals3*	0.3 ± 0.0	0.2 ± 0.0	0.3 ± 0.1	0.3 ± 0.0	0.2 ± 0.0	0.2 ± 0.0	0.2 ± 0.1	0.1 ± 0.0	0.1 ± 0.0	0.1 ± 0.0	0.1 ± 0.0	0.1 ± 0.0
*Lgals8*	1.4 ± 0.0	1.3 ± 0.1	1.4 ± 0.1	1.4 ± 0.0	1.8 ± 0.1	1.8 ± 0.0	1.9 ± 0.1	1.8 ± 0.1	1.5 ± 0.1	1.5 ± 0.1	1.4 ± 0.1	1.5 ± 0.0
*Lgals9*	0.5 ± 0.0	0.5 ± 0.1	0.5 ± 0.0	0.4 ± 0.1	0.2 ± 0.0	0.2 ± 0.0	0.2 ± 0.1	0.1 ± 0.0	0.3 ± 0.1	0.3 ± 0.0	0.3 ± 0.1	0.2 ± 0.0
*Tcfe3*	0.9 ± 0.1	0.8 ± 0.0	0.9 ± 0.1	0.9 ± 0.0	2.4 ± 0.4	2.4 ± 0.1	2.2 ± 0.3	2.4 ± 0.2	2.8 ± 0.1	2.7 ± 0.1	2.8 ± 0.0	2.9 ± 0.1
*Tcfeb*	0.9 ± 0.2	0.9 ± 0.1	0.8 ± 0.2	1.0 ± 0.2	1.0 ± 0.2	1.0 ± 0.1	0.9 ± 0.1	1.0 ± 0.2	0.8 ± 0.0	0.8 ± 0.1	0.7 ± 0.0	0.8 ± 0.0
*Tcfec*	0.8 ± 0.1	0.7 ± 0.0	0.8 ± 0.2	0.8 ± 0.1	0.9 ± 0.1	0.9 ± 0.0	0.9 ± 0.1	1.0 ± 0.1	0.8 ± 0.1	0.8 ± 0.1	0.8 ± 0.1	0.8 ± 0.1
*Mitf*	0.5 ± 0.1	0.5 ± 0.1	0.4 ± 0.1	0.4 ± 0.0	1.5 ± 0.3	1.4 ± 0.1	1.5 ± 0.2	1.5 ± 0.1	1.9 ± 0.2	1.9 ± 0.0	1.8 ± 0.2	1.9 ± 0.1
*Tgfb1*	0.6 ± 0.0	0.5 ± 0.0	0.6 ± 0.1	0.6 ± 0.0	0.4 ± 0.1	0.4 ± 0.0	0.3 ± 0.1	0.4 ± 0.1	0.4 ± 0.1	0.3 ± 0.0	0.4 ± 0.0	0.4 ± 0.1
*Tgfb3*	1.8 ± 0.1	1.6 ± 0.0	1.8 ± 0.2	1.8 ± 0.1	1.7 ± 0.0	1.7 ± 0.1	1.7 ± 0.2	1.7 ± 0.0	2.3 ± 0.0	2.3 ± 0.1	2.2 ± 0.0	2.3 ± 0.2
*Tgfbi*	0.2 ± 0.0	0.2 ± 0.1	0.2 ± 0.0	0.1 ± 0.0	0.4 ± 0.1	0.4 ± 0.0	0.4 ± 0.1	0.3 ± 0.1	0.3 ± 0.0	0.3 ± 0.0	0.3 ± 0.1	0.2 ± 0.0
*Tgfbr1*	1.7 ± 0.3	1.6 ± 0.1	1.7 ± 0.2	1.8 ± 0.1	3.1 ± 0.8	3.0 ± 0.4	3.2 ± 0.2	2.8 ± 0.3	2.2 ± 0.1	2.0 ± 0.1	2.3 ± 0.1	2.2 ± 0.0
*Tgfbr2*	0.8 ± 0.0	0.7 ± 0.0	0.7 ± 0.1	0.8 ± 0.2	0.6 ± 0.1	0.5 ± 0.1	0.5 ± 0.0	0.6 ± 0.0	0.7 ± 0.0	0.7 ± 0.1	0.6 ± 0.1	0.6 ± 0.0
*Tgfbr3*	1.3 ± 0.0	1.2 ± 0.0	1.3 ± 0.1	1.3 ± 0.0	1.1 ± 0.1	1.1 ± 0.1	1.2 ± 0.1	1.3 ± 0.1	1.9 ± 0.1	1.9 ± 0.0	1.9 ± 0.1	1.8 ± 0.1

## Data Availability

The data discussed in this publication have been deposited in NCBI’s Gene Expression Omnibus [33] and are accessible through GEO Series accession number GSE193583 (https://www.ncbi.nlm.nih.gov/geo/query/acc.cgi?acc=GSE193583, (accessed on 2 January 2022)).

## Supplementary Materials

The following are available online at https://www.mdpi.com/article/10.3390/ijms23031058/s1.
