# Supplementary material for: Cellular and Gene Expression Response to the Combination of Genistein and Kaempferol in the Treatment of Mucopolysaccharidosis Type I
Source: Int J Mol Sci. 2022 Jan 19;23(3):1058. doi: 10.3390/ijms23031058 (PMC8834790; doi:10.3390/ijms23031058)
Supplement: Supplementary file 1 [file ijms-23-01058-s001.zip › ijms-1534679-supplementary.pdf]

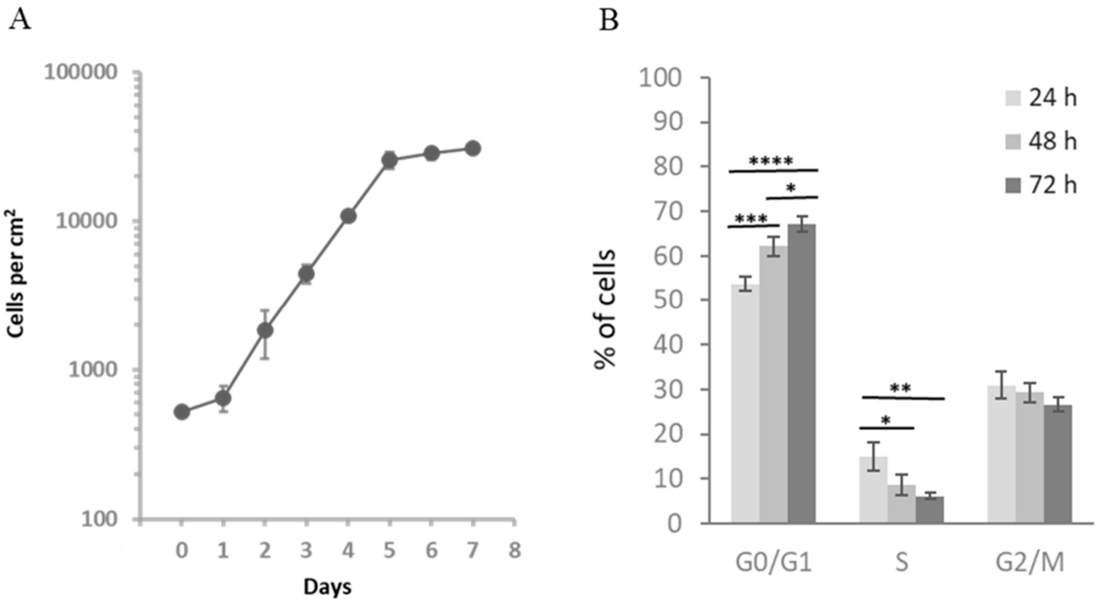

Figure S1. Growth curve for murine fibroblasts (A) seeded with an initial number of 5 x 10^4 per well of a 6-well plate (521 cells per cm^2). Data are presented as the mean cell count per cm^2 of growth well ± SD. Log phase of growth is observed between days 1 and 5. Doubling time calculated for cells in log phase = 18.11 h. Cell cycle profile of untreated mouse fibroblasts (control cells) in the log phase of growth (B). Cells were seeded in the same number as described above, incubated for 2 days until they entered the log phase of growth and culture medium was changed to fresh. Following an additional 24, 48 and 72 h of growth, cells were harvested and cell cycle profile was assessed using Muse® Cell cycle assay. Data are presented as mean ± SD. Asterisks indicate statistically significant differences (ANOVA with Tukey-Kramer HSD post hoc test, significance levels are indicated as * $p < 0.05$, ** $p < 0.01$, *** $p < 0.001$, and **** $p < 0.0001$).

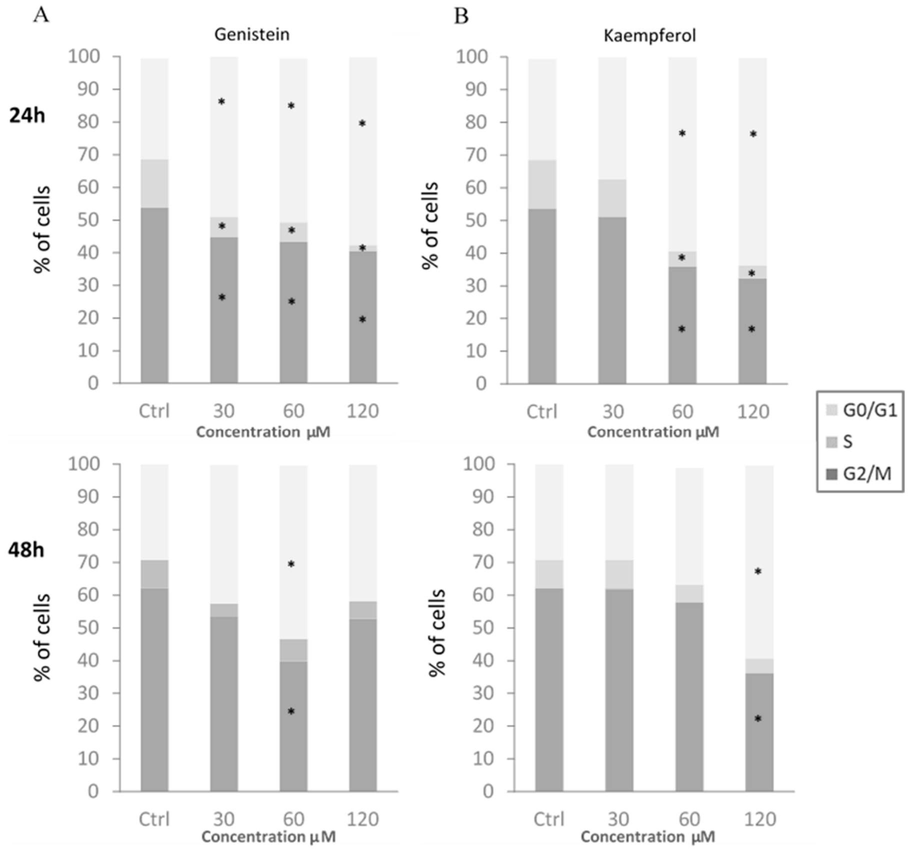

Figure S2. Cell cycle of murine fibroblasts after 24 h or 48 h exposure to genistein (A) and kaempferol (B) at a final concentration of 30, 60 or 120 µM. Ctrl—control cells treated with 0.1% DMSO. Data are presented as means of at least two biological repetitions. Most of the SD values are in the range of 1% to 3%; therefore, they are not shown in the charts but can be found in the Supplementary Table S1. An asterisk (*) indicates statistically significant differences compared to control cells (ANOVA with Tukey-Kramer HSD post hoc test, $p < 0.05$ or less).

Table S1. . Microarray expression profile of murine MPS I fibroblast genes coding for GAG metabolism proteins with expression profile modulated upon flavonoids treatment. Genes with up-regulated expression are marked in red, with down-regulated in blue.

GAG metabolism		Gene	Genistein		Kaempferol		Genistein + Kaempferol (1:1)	
			60 µM	120 µM	60 µM	120 µM	60 µM	120 µM
GAG synthesis	Chain initiation	*Xylt1*	1.3 ± 0.4					
		B4galt7			1.6 ± 0.2	1.4 ± 0.3	1.6 ± 0.1	
		Csgalnact1	1.7 ± 0.4	1.8 ± 0.0	3.0 ± 0.1	3.0 ± 0.5	3.2 ± 0.1	3.8 ± 0.2
		CSGALNACT2	1.3 ± 0.1	1.5 ± 0.1				
		Galnt5	0.6 ± 0.0		0.7 ± 0.0	0.7 ± 0.0		
	Chain elongation	*B3gnt2*			0.5 ± 0.1	0.4 ± 0.0	0.5 ± 0.0	0.5 ± 0.0
		B3gnt7					0.6 ± 0.1	
		B4galt1				0.7 ± 0.0	0.7 ± 0.1	0.6 ± 0.1
		B4galt3			1.4 ± 0.1	1.3 ± 0.1		1.3 ± 0.1
		B4GALT4	1.9 ± 0.0	2.2 ± 0.4	2.2 ± 0.4	3.3 ± 0.0	2.3 ± 0.1	2.6 ± 0.1
		B4galt5		0.7 ± 0.0	1.5 ± 0.2		1.4 ± 0.0	1.3 ± 0.1
		Chpf				0.7 ± 0.1	0.7 ± 0.0	0.7 ± 0.0
		Chpf2			1.6 ± 0.1	1.3 ± 0.0	2.3 ± 0.0	2.1 ± 0.0
		Chsy1		1.3 ± 0.1	1.4 ± 0.1	1.6 ± 0.3	1.8 ± 0.0	1.7 ± 0.0
		Ext1		0.7 ± 0.0	0.6 ± 0.1	0.6 ± 0.0	0.5 ± 0.0	0.6 ± 0.1
		Extl3		1.6 ± 0.0	1.3 ± 0.1	1.6 ± 0.1		1.4 ± 0.1
		Has1			0.7 ± 0.1		0.5 ± 0.1	0.7 ± 0.1
		Has2		1.3 ± 0.2	0.6 ± 0.1	0.5 ± 0.1	0.5 ± 0.0	0.5 ± 0.1
		Has3				1.3 ± 0.2		
		Hs3st1		1.5 ± 0.0	2.3 ± 0.2	4.4 ± 0.5	2.3 ± 0.3	3.2 ± 0.8
		Hs3st2				1.3 ± 0.4	0.6 ± 0.0	0.5 ± 0.0
		Hs6st2	0.7 ± 0.0	0.5 ± 0.1	0.5 ± 0.0	0.3 ± 0.1	0.6 ± 0.2	0.4 ± 0.0
		Hs6st3	0.6 ± 0.1	0.5 ± 0.1	0.5 ± 0.1	0.3 ± 0.0	0.5 ± 0.1	0.5 ± 0.0
		ST3GAL1		0.6 ± 0.1		0.7 ± 0.1		
		ST3GAL2	1.3 ± 0.0	1.6 ± 0.5				
		ST3GAL3	1.5 ± 0.0	1.7 ± 0.2			1.3 ± 0.1	1.3 ± 0.1
	Chain modification	*Chst1*	0.7 ± 0.0					0.7 ± 0.0
		Chst11	1.3 ± 0.0	2.2 ± 0.4	2.0 ± 0.1	2.4 ± 0.3	2.2 ± 0.4	2.5 ± 0.1
		Chst12	0.7 ± 0.0	0.6 ± 0.1	0.6 ± 0.0	0.6 ± 0.0	0.7 ± 0.0	0.7 ± 0.0
		Chst15		0.7 ± 0.2	1.6 ± 0.1	1.3 ± 0.3	1.6 ± 0.5	1.3 ± 0.0

	Chst2		2.0 ± 0.2	2.0 ± 0.0	2.5 ± 0.3	2.6 ± 0.2	3.1 ± 0.0
	Chst3					2.1 ± 0.5	1.6 ± 0.1
	Chst7		1.3 ± 0.0				
	Chst8	2.2 ± 0.1			1.4 ± 0.4	0.3 ± 0.0	0.3 ± 0.1
	Dse		0.7 ± 0.0	0.6 ± 0.0	0.5 ± 0.0	0.6 ± 0.0	0.5 ± 0.0
	Dsel		0.6 ± 0.0				
	Fut8		0.7 ± 0.0				
	Ndst2		0.7 ± 0.1	0.7 ± 0.0	0.6 ± 0.0	0.6 ± 0.0	0.6 ± 0.0
	Ndst3		0.7 ± 0.0	0.6 ± 0.2		0.7 ± 0.2	
	Ndst4	1.5 ± 0.2			1.3 ± 0.1	1.3 ± 0.0	
	St3gal1		0.6 ± 0.1		0.7 ± 0.1		
	St3gal2	1.3 ± 0.0	1.6 ± 0.5				
	St3gal3	1.5 ± 0.0	1.7 ± 0.2			1.3 ± 0.1	1.3 ± 0.0
	Glce				1.5 ± 0.1		1.4 ± 0.1
	Hs3st3a1	0.6 ± 0.2	0.5 ± 0.1	0.6 ± 0.0	0.4 ± 0.0	0.6 ± 0.3	0.6 ± 0.1
	Hs3st3b1		1.7 ± 0.3			1.4 ± 0.0	1.5 ± 0.1
	Ust			0.5 ± 0.0	0.6 ± 0.0	0.6 ± 0.1	0.6 ± 0.0
**GAG degradation**	*Arsb*				0.7 ± 0.0		
	Glb1l					1.6 ± 0.0	1.3 ± 0.1
	Gns	1.4 ± 0.0					
	Gusb	0.7 ± 0.1	0.5 ± 0.0				
	Hexb	1.5 ± 0.0	1.7 ± 0.1		1.5 ± 0.0	1.6 ± 0.0	1.8 ± 0.0
	Hgsnat	1.5 ± 0.1	1.8 ± 0.1	1.4 ± 0.3	1.5 ± 0.1	1.3 ± 0.2	1.4 ± 0.1
	Hpse2					0.5 ± 0.1	0.7 ± 0.1
	Hyal1	2.4 ± 0.2	4.4 ± 0.1	2.2 ± 0.2	2.7 ± 0.3	4.2 ± 0.4	4.6 ± 0.1
	Ids	1.3 ± 0.1	1.4 ± 0.1				
	Naglu	1.3 ± 0.3	1.4 ± 0.1	1.4 ± 0.0	1.5 ± 0.1	1.5 ± 0.0	1.5 ± 0.2
	Sgsh	1.3 ± 0.0	1.4 ± 0.2			1.3 ± 0.0	1.4 ± 0.0
**GAG-related metabolism**	*Abcc5*	1.4 ± 0.2	1.4 ± 0.1	1.3 ± 0.1	1.3 ± 0.1		
	Agrn		1.8 ±0.1	1.6 ± 0.3	2.1 ± 0.4	2.3 ± 0.2	2.3 ± 0.3
	Angpt1		0.4 ± 0.1	0.4 ± 0.1	0.2 ± 0.0	0.6 ± 0.1	0.5 ± 0.2
	Cemip		0.3 ± 0.1		0.4 ± 0.0		
	Cspg4	0.7 ± 0.0	0.5 ± 0.0		0.7 ± 0.0	0.7 ± 0.0	0.6 ± 0.1
	Cspg5	1.4 ± 0.1	1.5 ± 0.1				
	Cytl1					0.6 ± 0.1	0.5 ± 0.1

	Fmod	0.6 ± 0.1	0.4 ± 0.0	0.6 ± 0.0	0.5 ± 0.0		0.7 ± 0.0
	Gal3st3	1.8 ± 0.4		1.4 ± 0.1			1.3 ± 0.1
	Gal3st4						1.4 ± 0.3
	Gcnt2	0.5 ± 0.0	0.7 ± 0.1		0.7 ± 0.1	0.7 ± 0.1	0.7 ± 0.1
	Gpc1	0.6 ± 0.1	0.6 ± 0.1	1.4 ± 0.0	1.3 ± 0.0		0.7 ± 0.0
	Gpc2	1.7 ± 0.3	2.1 ± 0.4	2.7 ± 0.2	4.4 ± 0.5	2.0 ± 0.1	2.3 ± 0.4
	Gpc3						1.3 ± 0.0
	Gpc4			0.4 ± 0.0	0.3 ± 0.0	0.5 ± 0.0	0.5 ± 0.1
	Gpc6		0.5 ± 0.0		0.5 ± 0.1		
	Hspg2	0.7 ± 0.1	0.6 ± 0.1	0.7 ± 0.2	0.7 ± 0.0	0.5 ± 0.0	0.6 ± 0.1
	Ogn	1.3 ± 0.1	0.7 ± 0.1	0.5 ± 0.0	0.3 ± 0.0	0.5 ± 0.1	0.5 ± 0.0
	Omd			0.3 ± 0.0	0.2 ± 0.0	0.4 ± 0.1	0.3 ± 0.1
	Pdgfrb		0.6 ± 0.0			0.7 ± 0.0	0.7 ± 0.0
	Prelp	1.5 ± 0.1	1.5 ± 0.2	0.6 ± 0.1	0.7 ± 0.0		
	Ptger4		1.4 ± 0.0	1.4 ± 0.2	1.4 ± 0.1	1.7 ± 0.4	1.7 ± 0.1
	Pxylp1	1.5 ± 0.2		1.3 ± 0.4		1.5 ± 0.3	
	Sdc2					1.3 ± 0.0	1.5 ± 0.1
	Sdc3	0.7 ± 0.1	0.5 ± 0.1	0.5 ± 0.0	0.4 ± 0.0	0.3 ± 0.0	0.3 ± 0.0
	Sdc4	0.7 ± 0.0		0.3 ± 0.1	0.2 ± 0.1	0.5 ± 0.0	0.4 ± 0.1
	Slc35d1		1.8 ± 0.3				
	Sulf1	0.7 ± 0.0	0.5 ± 0.0	0.5 ± 0.0	0.3 ± 0.1	0.3 ± 0.1	0.3 ± 0.1
	Sulf2	1.7 ± 0.2	2.0 ± 0.1	1.7 ± 0.3	1.8 ±0.1	1.9 ± 0.2	2.3 ± 0.1
	Tgfb1	0.4 ± 0.1	0.6 ± 0.0	0.5 ± 0.1	0.4 ± 0.1	0.4 ± 0.1	0.4 ± 0.1
	Tpst1		0.6 ± 0.0		0.7 ± 0.0		
	Tpst2		1.9 ± 0.0				
	Vcan		1.4 ± 0.0		1.4 ± 0.1		1.3 ± 0.0

Table S2. Microarray analysis of genes with modulated expression coding for molecular factors involved in Cell cycle phases. Down-regulated genes are denoted in blue, up-regulated in red. Numbers in brackets stands for number of modulated genes in particular Cell cycle phase.

Cell cycle phase	Genistein		Kaempferol		Genistein + Kaempferol (1:1)	
	60 μM	120 μM	60 μM	120 μM	60 μM	120 μM
G1	Cdk6, Myc (2)<br><br>Ccnd2, E2f1, Rb1, Rbl2 (4)	Anapc11, Cdk2, Cdk6 (3)<br><br>Anapc7, Ccnd1, Ccnd2, E2f1, Rb1, Rbl2 (6)	Cdk6 (1)<br><br>Abl1, Ccnd2, Rb1, Rbl2 (4)	Cdk6 (1)<br><br>Anapc7, Ccnd2, Cdk4, Rb1 (4)	Cdh1, Cdk6 (2)<br><br>Anapc13, Anapc7, Ccnd2, E2f1, Rb1, Rbl2 (6)	Ccnd3, Cdh1, Cdk6 (3)<br><br>Abl1, Anapc13, Anapc7, Ccnd2, E2f1, Rb1, Rbl1, Rbl2 (8)
G1/S	Ccne1, Ccne2, Cdc6, Cdk6, Cdkn2d, Gadd45b, Hdac,Mcm5, Mcm6, Myc, Skp2, Tgfb1 (12)<br><br>Cdkn1a, Cdkn2b, Cdkn2c, Chek2, E2f1, Gadd45a, Hdac8, Rb1, Rbl2, Smad3, Tfdp2, Tgfb3 (12)	Ccne1, Ccne2, Cdc20, Cdc6, Cdk1, Cdk2, Cdk6, Cdkn1b, Cdkn2c, Cdkn2d, Chek1, Fzr1, Hdac7, Mcm10, Mcm3,Mcm5, Mcm6, Pcna, Skp2, Tgfb1, Tgfb2, Wee1 (22)<br><br>Cdkn1a, Chek2, E2f1, Gadd45a, Gadd45b, Hdac4, Hdac8, Mdm2, Rb1, Rbl2, Tgfb3 (11)	Ccne1, Ccne2, Cdk6, Cdkn2d, Tgfb1 (5)<br><br>Cdc45, Cdc7, Cdkn1a, Cdkn2b, Chek2, Gadd45a, Hdac4, Hdac6, Mcm7, Mcm8, Mdm2, Rb1, Rbl2, Tfdp2, Tgfb3, Wee1 (16)	Ccne1, Ccne2, Cdc20, Cdk1, Cdk6, Cdkn2d, E2f4, Hdac7, Mcm6, Skp2, Tgfb1 (11)<br><br>Cdkn1a, Cdkn2b, Chek2, Crebbp, Gadd45a, Hdac4, Mdm2, Rb1, Tfdp2, Tgfb3 (10)	Cdk6, Tgfb1 (2)<br><br>Abl1, Cdc45, Cdc7, Cdkn1a, Cdkn2b, Cdkn2c, Chek2, E2f1, E2f2, Gadd45a, Hdac4, Mcm10, Mcm3, Mcm4, Mcm7, Mcm8, Mdm2, Orc1, Rb1, Rbl2, Sfn, Tfdp2, Tgfb3 (23)	Ccne1, Ccne2, Cdk6, E2f4, Gadd45b, Myc, Tgfb1 (7)<br><br>Cdc45, Cdc7, Cdkn1a, Cdkn1b, Cdkn2b, Cdkn2c, Chek2, E2f1, E2f2, E2f5, E2f6, Gadd45a, Hdac4, Mcm8, Mdm2, Rb1, Rbl1, Rbl2, Tfdp2, Tgfb3, Wee1 (21)
S	Myc, Skp2 (2)<br><br>Kitl, Prkdc, Rb1, Rbl2, Smad3 (5)	Cdk2, Dbf4, Men1, Skp2 (4)<br><br>Prkdc, Rb1, Rbl2 (3)	Kitl, Ube2f (2)<br><br>Mpeg1, Prkdc, Rb1, Rbl2 (4)	Dbf4, Kitl, Skp2 (3)<br><br>Mpeg1, Prkdc, Rb1 (3)	Ep300, Ube2f (2)<br><br>Men1,Mpeg1, Prkdc, Rb1, Rbl2 (5)	Ep300, Myc, Ube2f (3)<br><br>Mpeg1, Prkdc, Rb1, Rbl1, Rbl2 (5)
S/G2	(0)<br><br>Chek2, Prkdc (2)	Ccna2, Cdk2, Chek1, Dbf4 (4)<br><br>Chek2, Prkdc (2)	(0)<br><br>Chek2, Prkdc (2)	Ccna2, Dbf4 (2)<br><br>Chek2, Prkdc (2)	(0)<br><br>Chek2, Prkdc (2)	Ccna2 (1)<br><br>Ccna1, Chek2, Prkdc (3)
G2	Gadd45b (1)<br><br>E2f1, Gadd45a, Mdm2, Prkdc (4)	Fzr1, Wee1 (2)<br><br>E2f1, Gadd45a, Gadd45b, Mdm2, Prkdc (5)	(0)<br><br>Gadd45a, Mdm2, Prkdc, Wee1 (4)	(0)<br><br>Gadd45a, Mdm2, Prkdc (3)	(0)<br><br>E2f1, E2f2, Gadd45a, Mdm2, Prkdc (5)	Gadd45b (1)<br><br>E2f1, E2f2, Gadd45a, Mdm2, Prkdc, Wee1 (6)

G2/M	Cdc25c, Cdc6, Gadd45b, Myc, Plk1, Ywhah (6)	Ccna2, Ccnb1, Ccnb2, Cdc20, Cdc25b, Cdc25c,Cdc6, Cdk1, Chek1, Pkmyt1, Plk1, Wee1, Ywhah (13)	Plk1, Wee2, Ywhah (3)	Ccna2, Ccnb1, Cdc20, Cdc25b, Cdc25c, Cdk1, Pkmyt1, Plk1, Ywhab, Ywhah (10)	Plk1, Wee2, Ywhah (3)	Ccna2, Gadd45b, Myc, Plk1, Wee2, Ywhah (6)
	Ccnb3, Chek2, Gadd45a, Mdm2, Rb1, Rbl2, Wee2 (7)	Chek2, Gadd45a, Gadd45b, Mdm2, Rb1, Rbl2 (6)	Atm, Atr, Chek2, Gadd45a, Mdm2, Rb1, Rbl2, Wee1 (8)	Chek2, Gadd45a, Mdm2, Rb1 (4)	Atr, Chek2, Gadd45a, Mdm2, Mpeg1, Pkmyt1, Rb1, Rbl2, Sfn (9)	Atr, Ccna1, Chek2, Gadd45a, Mdm2, Rb1, Rbl1, Rbl2, Wee1 (9)
M	Cdc25c, Stag1 (2)	Anapc11, Bub1, Bub1b, Ccna2, Ccnb1, Ccnb2, Cdc25b, Cdc25c, Espl1, Mad2l1, Rad21, Stag1, Ttk (13)	(0)	Ccna2, Ccnb1, Cdc25b, Cdc25c, Mad2l1, Stag1 (6)	Cdh1, Ep300, Espl1, Stag1 (4)	Ccna2, Cdh1, Ep300, Stag1 (4)
	Ccnb3, Mdm2, Pttg1, Stag2 (4)	Anapc7, Cdc14a, Mdm2, Pttg1, Stag2 (5)	Atm, Atr, Cdc23, Mad1l1, Mdm2, Pttg1, Ttk (7)	Anapc7, Crebbp, Mad1l1, Mdm2, Pttg1, Tbc1d8 (6)	Anapc13, Anapc7, Atr, Mad1l1, Mdm2, Pttg1, Tbc1d8 (7)	Anapc13, Anapc7, Atr, Ccna1, Cdc14a, Mad1l1, Mdm2, Pttg1, Tbc1d8 (9)

Table S3. Microarray analysis of murine MPSI fibroblast genes coding for lysosomal proteins with modulated expression upon treatment with tested flavonoids

Enzyme	Gene	Genistein		Kaempferol		Genistein + Kaempferol (1:1)	
		60 µM	120 µM	60 µM	120 µM	60 µM	120 µM
Glycosidases	*Gla*	1.5 ± 0.1	1.7 ± 0.0	1.7 ± 0.3	2.1 ± 0.4	1.6 ± 0.1	1.8 ±0.1
	Glb1l					1.6 ± 0.0	1.3 ± 0.1
	Gusb		0.5 ± 0.0				
	Fuca1	1.8 ±0.2	2.5 ± 0.0	1.7 ± 0.1	1.6 ± 0.2	2.2 ± 0.0	2.1 ± 0.0
	Hexb	1.5 ± 0.0	1.7 ± 0.1	1.2 ± 0.0	1.5 ± 0.1	1.6 ± 0.0	1.8 ± 0.0
	Hexdc		1.5 ± 0.0	1.4 ± 0.2		2.2 ± 0.6	2.0 ± 0.0
	Hpse2					0.5 ± 0.1	0.7 ± 0.1
	Hyal1	2.4 ± 0.2	4.4 ± 0.1	2.2 ± 0.2	2.7 ± 0.3	4.2 ± 0.4	4.6 ± 0.1
	Manba	1.3 ± 0.2					
	Man2b1	1.7 ± 0.2	1.8 ± 0.4		1.3 ± 0.0	1.5 ± 0.1	1.5 ± 0.1
	Man2c1				0.6 ± 0.0		
	Naga			0.7 ± 0.0	0.7 ± 0.1		
	Naglu	1.3 ± 0.3	1.4 ± 0.1	1.4 ± 0.0	1.5 ± 0.1	1.5 ± 0.0	1.5 ± 0.2
	Neu1	2.1 ± 0.2	3.0 ± 0.7	1.6 ± 0.1	1.8 ±0.1	2.2 ± 0.2	2.3 ± 0.2
	Neu2					0.7 ± 0.1	
	Neu4					0.5 ± 0.1	0.6 ± 0.0
Sulfatase	*Arsa*					1.7 ± 0.3	1.5 ± 0.2
	Arsb				0.7 ± 0.0		
	Gns	1.4 ± 0.0					
	Ids	1.3 ± 0.1	1.4 ± 0.1				
	Sgsh	1.3 ± 0.0	1.4 ± 0.2			1.3 ± 0.0	1.4 ± 0.0
Ceramidase	*Asah1*	1.4 ± 0.1					
Sfingomylinases	*Smpd1*			0.6 ± 0.1	0.7 ± 0.0		
Other lysosomal enzymes and activators	*Aga*	2.0 ± 0.1	2.4 ± 0.1	1.6 ± 0.1	2.2 ± 0.2	2.0 ± 0.1	2.2 ± 0.0
	Gm2a	1.6 ± 0.4				1.7 ± 0.3	1.5 ± 0.1
	Gnptg	1.7 ± 0.0	1.9 ± 0.2		1.3 ± 0.3		
	Nagpa				0.6 ± 0.1	0.7 ± 0.0	0.5 ± 0.0
	Ppt1	1.9 ± 0.1	2.3 ± 0.4			1.4 ± 0.0	1.5 ± 0.1
	Ppt2		1.3 ± 0.3		1.5 ± 0.0	1.5 ± 0.2	1.5 ± 0.1
	Psapl1					0.6 ± 0.0	
Lipases	*Lipa*			1.3 ± 0.0		1.4 ± 0.1	1.4 ± 0.2
Proteases	*Ctsb*	1.3 ± 0.1	1.3 ± 0.1				
	Ctsd	1.4 ± 0.1	1.5 ± 0.1	1.3 ± 0.0	1.3 ± 0.1	1.8 ± 0.2	1.8 ± 0.0
	Ctsf	1.8 ± 0.2	2.3 ± 0.1	1.5 ± 0.2	1.7 ± 0.0	1.7 ± 0.0	1.9 ± 0.1
	Ctsg					0.6 ± 0.1	0.6 ± 0.0
	Ctsh			1.4 ± 0.2		1.4 ± 0.1	
	Ctsk			1.8 ± 0.2	1.7 ± 0.1	1.5 ± 0.2	1.3 ± 0.1
	Ctsl			1.3 ± 0.0	1.5 ± 0.1	1.2 ± 0.1	1.2 ± 0.0
	Ctso	1.3 ± 0.0	1.3 ± 0.2	0.7 ± 0.1		1.2 ± 0.0	
	Tpp1	1.5 ± 0.1	1.6 ± 0.1			1.3 ± 0.0	1.3 ± 0.1
Lysosomal membrane proteins	*Abca2*					1.5 ± 0.2	1.7 ± 0.2
	Abca9		0.4 ± 0.1	0.4 ± 0.1	0.3 ± 0.1	0.4 ± 0.1	0.5 ± 0.0
	Cd68	3.7 ± 0.0	8.3 ± 0.5	1.6 ± 0.1	1.3 ± 0.4	1.7 ± 0.2	1.5 ± 0.2
	Cln3	1.4 ± 0.0		1.4 ± 0.0	1.6 ± 0.1		
	Cln5					1.3 ± 0.1	1.3 ± 0.1

	Ctns	1.7 ± 0.2	1.7 ± 0.2	1.8 ± 0.1	2.3 ± 0.1	1.7 ± 0.0	1.8 ± 0.0
	Entpd4				0.7 ± 0.0		
	Entpd4				0.7 ± 0.0		
	Hgsnat	1.5 ± 0.1	1.8 ± 0.1	1.4 ± 0.3	1.5 ± 0.1	1.3 ± 0.1	1.4 ± 0.1
	Lamp1						
	Lamp2	1.3 ± 0.1					
	Laptm4b	1.5 ± 0.1	1.4 ± 0.1	1.3 ± 0.0		1.3 ± 0.1	1.3 ± 0.0
	Laptm5					0.6 ± 0.1	0.6 ± 0.0
	Mcoln1	2.1 ± 0.4	2.8 ± 0.4	2.0 ± 0.1	2.7 ± 0.1	2.4 ± 0.1	2.2 ± 0.0
	Mfsd8	1.5 ± 0.1	1.4 ± 0.1				1.4 ± 0.0
	Npc1	1.4 ± 0.0	1.7 ± 0.2	1.6 ± 0.1	2.0 ± 0.2	1.4 ± 0.1	1.4 ± 0.0
	Npc2	1.6 ± 0.3	1.6 ± 0.2	1.4 ± 0.0	1.6 ± 0.3	1.7 ± 0.1	1.7 ± 0.0
	Scarb2		1.7 ± 0.1				
	Slc17a5	1.5 ± 0.0	1.9 ± 0.4	1.9 ± 0.4	2.4 ± 0.1	1.9 ± 0.1	2.0 ± 0.1
	Slc11a1						
	Slc11a2		1.5 ± 0.2	1.3 ± 0.3		1.5 ± 0.2	1.5 ± 0.2
	Sort1	1.8 ± 0.0	1.9 ± 0.1	2.0 ± 0.3	2.1 ± 0.2	3.0 ± 0.1	3.6 ± 0.4
	Atp6ap1l				0.7 ± 0.3		
	Tcirg1 (ATP6V0A3)			1.3 ± 0.0	1.4 ± 0.1		
	Atp6v0a1					1.4 ± 0.0	1.4 ± 0.1
	Atp6v0a2				1.3 ± 0.0		
	Atp6v0a4	1.5 ± 0.3					0.7 ± 0.2
	Atp6v0b	1.3 ± 0.5	1.6 ± 0.2		1.3 ± 0.1	1.9 ± 0.0	2.0 ± 0.1
	Atp6v0d2	0.6 ± 0.1	0.6 ± 0.1	0.6 ± 0.0	0.6 ± 0.0	0.5 ± 0.1	0.5 ± 0.1
	Atp6v0e		1.5 ± 0.2			1.4 ± 0.0	1.3 ± 0.2
	Atp6v0e2	1.9 ± 0.2	2.6 ± 0.2	1.5 ± 0.0	1.7 ± 0.2	1.6 ± 0.0	2.1 ± 0.1
V-ATPases	*Atp6v1a*	1.9 ± 0.2	2.0 ± 0.0	1.3 ± 0.1		1.5 ± 0.1	1.5 ± 0.1
	Atp6v1b1						
	Atp6v1b2	1.4 ± 0.0	1.6 ± 0.0			1.3 ± 0.1	1.4 ± 0.0
	Atp6v1d	1.5 ± 0.0	1.8 ± 0.1	1.4 ± 0.1	1.4 ± 0.0	1.3 ± 0.0	1.3 ± 0.1
	Atp6v1e1	1.3 ± 0.0	1.5 ± 0.1			1.6 ± 0.1	1.5 ± 0.1
	Atp6v1e2	1.4 ± 0.1	1.3 ± 0.3				
	Atp6v1f		1.5 ± 0.1			1.3 ± 0.0	
	Atp6v1g1		1.4 ± 0.1				
	Atp6v1g2					1.6 ± 0.6	1.4 ± 0.2
	Atp6v1g3			0.7 ± 0.2			
	Atp6v1h		1.5 ± 0.1				
